# Biodiversity data integration—the significance of data resolution and domain

**DOI:** 10.1371/journal.pbio.3000183

**Published:** 2019-03-18

**Authors:** Christian König, Patrick Weigelt, Julian Schrader, Amanda Taylor, Jens Kattge, Holger Kreft

**Affiliations:** 1 Biodiversity, Macroecology & Biogeography, University of Goettingen, Goettingen, Germany; 2 Research Group Functional Biogeography, Max Planck Institute for Biogeochemistry, Jena, Germany; 3 German Centre for Integrative Biodiversity Research (iDiv) Halle-Jena-Leipzig, Leipzig, Germany; 4 Centre of Biodiversity and Sustainable Land Use (CBL), University of Goettingen, Goettingen, Germany; University College London, UNITED KINGDOM

## Abstract

Recent years have seen an explosion in the availability of biodiversity data describing the distribution, function, and evolutionary history of life on earth. Integrating these heterogeneous data remains a challenge due to large variations in observational scales, collection purposes, and terminologies. Here, we conceptualize widely used biodiversity data types according to their domain (what aspect of biodiversity is described?) and informational resolution (how specific is the description?). Applying this framework to major data providers in biodiversity research reveals a strong focus on the disaggregated end of the data spectrum, whereas aggregated data types remain largely underutilized. We discuss the implications of this imbalance for the scope and representativeness of current macroecological research and highlight the synergies arising from a tighter integration of biodiversity data across domains and resolutions. We lay out effective strategies for data collection, mobilization, imputation, and sharing and summarize existing frameworks for scalable and integrative biodiversity research. Finally, we use two case studies to demonstrate how the explicit consideration of data domain and resolution helps to identify biases and gaps in global data sets and achieve unprecedented taxonomic and geographical data coverage in macroecological analyses.

## Abstract 2

This Essay highlights data resolution as central property of biodiversity data that affects the precision and representativeness of macroecological inferences. It also discusses ways to maximize synergies among data types and showcases the potential of cross-resolution, cross-domain data integration.

The biosphere is facing unprecedented pressure from habitat loss, climate change, and the introduction of nonnative species [1–3]. To better understand how biodiversity will be affected under changing environmental conditions, data from multiple ecological disciplines have to be integrated across a wide range of spatiotemporal scales [4,5]. Significant progress towards this objective has been made in recent years. Initiatives such as the Global Biodiversity Information Facility (GBIF) [6], TRY [7], sPlot [8], and GenBank [9] provide access to massive collections of biological data that drive increasingly comprehensive macroecological analyses [10–12]. At the same time, it is becoming clearer that the naïve accumulation of evermore data will not resolve the widespread gaps and biases in ecological data sets [13–15]. A more systematic understanding of biodiversity data types, their applications, and their synergetic potentials is needed. Focusing on vascular plants, our aim here is to help build such an understanding in order to improve the coverage, representativeness, and usefulness of global biodiversity data.

## Data domains, types, and resolutions

Biodiversity research is organized into domains that cover distinct spheres of knowledge, e.g., of the taxonomy, geographical distribution, or functional traits of organisms [16]. For an effective integration and utilization of biodiversity data, two domains are of particular importance. Biogeography, on the one hand, studies the distribution of life across space and time [17], providing a key link between organisms and their environment. Biogeographical data can therefore be linked to a wide range of organismic (e.g., taxonomic, functional, phylogenetic) and environmental (e.g., climate, soil, topography) information. Functional ecology, on the other hand, aims to approximate the ecological strategy of organisms by means of measurable traits that vary across species [18] and offers a mechanistic approach to understanding ecological patterns and processes. Functional biogeography—the combination of these two domains—allows for the systematic study of trait variation along biotic and abiotic gradients at different scales and represents a promising approach to build a mechanistic understanding of plant diversity [19]. For these reasons, we focus our discussion on the key domains of biogeography and functional ecology.

**Fig 1:**
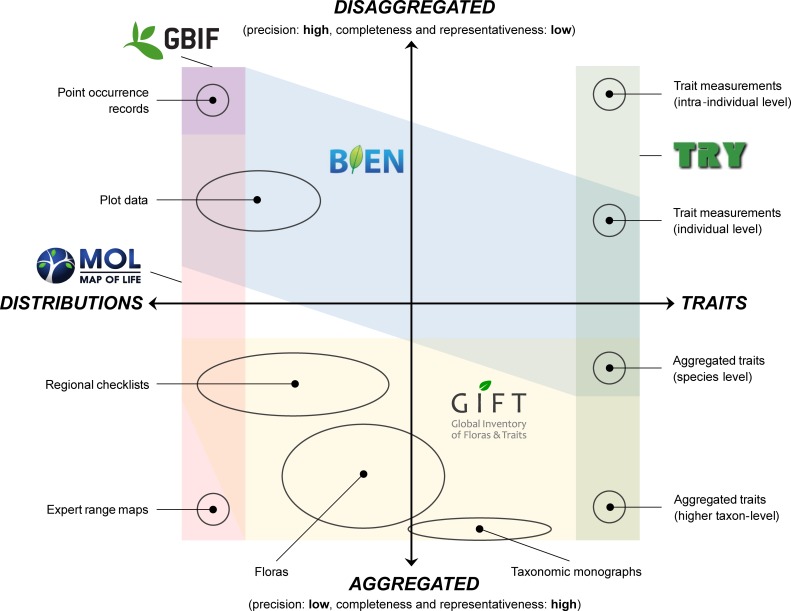
Selected biodiversity data types, arranged according to their primary domain (here, species distributions versus functional traits) and informational resolution (disaggregated versus aggregated). Projects that integrate global plant diversity data are often domain-specific (e.g., Map of Life [24]; TRY [7]) or focus on the disaggregated end of the data spectrum (e.g., GBIF [6], BIEN [26]). Complementing the ecological data landscape with aggregated data (e.g., GIFT [28]) creates strong synergies and facilitates biodiversity data integration across domains and resolutions. BIEN, Botanical Information Network and Ecology Network; GBIF, Global Biodiversity Information Facility; GIFT, Global Inventory of Floras and Traits.

A domain is typically associated with a set of domain-specific data types (Fig 1). Species distributions, e.g., can be represented by point occurrences, vegetation plots, checklists, or range maps. Functional trait data may come as field measurements for individual organisms or as aggregated values for populations, species, higher taxa (e.g., genera, families) or functional groups (e.g., plant functional types). Additionally, some biodiversity data types combine information from multiple domains, e.g., regional Floras representing sources of both distributional and functional information.

Biodiversity data types provide information at varying resolutions. Although the concept of resolution has substantially improved our understanding of spatial biodiversity patterns [20,21], it is less commonly used in other contexts. However, resolution is a general property of biodiversity data that can be understood as the degree of ecological generalization represented by a given data type. Highly disaggregated data, e.g., point occurrences or trait measurements, represent a single sampling event for a particular individual at a certain location and time. In contrast, highly aggregated data, e.g., Floras or taxonomic monographs, provide a more general account of biodiversity across large spatial, temporal, and taxonomic scales. There is a fundamental trade-off between fine-scale precision and large-scale representativeness across the data resolution spectrum. Although disaggregated data provide the necessary detail to address questions at the level of populations or communities [8,22], they tend to be less complete and representative at macroecological scales [13,15]. Aggregated data, on the other hand, are limited in their capacity to resolve fine-grained ecological patterns but usually provide higher completeness and representativeness at large scales. This trade-off, which, too, has been mostly described in geographical contexts [23,24], is highly relevant for the precision and accuracy of macroecological inferences [16,25].

Most projects for the integration of biodiversity data focus on the disaggregated end of the data spectrum (e.g., GBIF [6], Botanical Information Network and Ecology Network (BIEN) [26], sPlot [29], or TRY [7]). Given the above-mentioned trade-offs and their implications for large-scale coverage and representativeness of biodiversity data, a stronger consideration of aggregated data (e.g., World Checklist of Selected Plant families [WCSP] [27] or Global Inventory of Floras and Traits [GIFT] [28]) seems instrumental for establishing robust global baselines in plant diversity research. This not only opens up new opportunities but also poses new challenges with respect to data collection, mobilization, and sharing, as well as the utilization of synergies across data types.

## Data collection and processing

The integration of biodiversity data starts in the field, with the primary data collected in surveys, experiments, and other research efforts. Such data are usually tailored to answer a particular research question. Therefore, robust ecological generalizations require large quantities of disaggregated or aggregated data that are organized and integrated in biodiversity databases. The quality and coverage of such databases can be greatly improved when primary research projects put strong emphasis on the utility and reusability of collected data for secondary scientific purposes [30].

The utility of primary data for data integration efforts can be increased in several ways. First, focusing on regions, ecosystems, plant groups, or functional traits that are currently underrepresented in global biodiversity databases increases the general interest in the collected data and the study itself. Coverage analyses based on integrated biodiversity resources can provide guidance by identifying knowledge gaps and setting research priorities [15]. Second, cross-institutional coordination of research projects creates synergies through standardized methods and complementary research foci. Research networks such as International Long Term Ecological Research network (ILTER) [31] provide an ideal framework to utilize these synergies [32]. Third, an efficient study design helps to maximize the data output given the available resources. This can be aided, e.g., by optimizing study logistics and surveying effort [33], applying power analyses to estimate required sample sizes [34], and cooperating closely with local field guides and botanists [35]. Throughout the process of data collection, digital solutions such as Open Data Kit [36] can help to conveniently enter, cross-check, annotate, and aggregate field data. This increases data integrity and provides crucial meta-information for subsequent quality assessments and integration efforts.

The reusability of primary data can be ensured by adopting existing data standards and protocols. Species names—the most critical common identifier for data integration—can be standardized using pertinent software packages [37,38], which spell-check input names and match them against authoritative taxonomic resources. Moreover, trait measurement protocols [39] and terminologies [40] facilitate interoperability across research projects. The exchange of diversity data is supported by open data standards like Darwin Core [41] or Humboldt Core [42]. Finally, innovative publishing frameworks, such as the Biodiversity Data Journal [43] or the GBIF Integrated Publishing Toolkit [6], allow for a quick publication of standardized, annotated, and easily accessible data sets.

## Data mobilization

A prime example of successful data mobilization is the massive extraction of distributional information from preserved specimens within the last 15 years. However, specimen records hold other types of information as well [44]. In particular, the (semi-)automated extraction of traits from herbarium specimens represents an area of largely unused potential. Standardized measurements on collected plant material may be incorporated into digitization workflows, potentially yielding thousands of geographically defined records of, e.g., specific leaf area [45] or phenological plant information [46]. Also, images of already digitized specimens can be used to retrieve functional traits [47]. Nonetheless, the set of traits that can be (nondestructively) obtained from herbarium specimens excludes many important characteristics, e.g., plant growth form, vegetative height, or stem specific density.

Another way to mobilize substantial amounts of ecological data—mainly from the aggregated end of the data spectrum—lies in botanical literature, e.g., Floras, checklists, and taxonomic monographs. Such resources are broadly available [48] and provide expert-validated distributional information, often including the biogeographical status of the species listed (e.g., endemic, native, introduced). Moreover, descriptions of morphology, life history, flowers, fruits, seeds, phenology, and other functionally relevant features are often available. Considering the wealth of information contained in published floristic literature, the development of general, scalable methods for data extraction seems to be central for improving the coverage of biodiversity databases. First projects that implement such workflows show promising results [49,50] and could contribute significantly to gap-filling of primary biodiversity data.

## Data imputation

**Fig 2:**
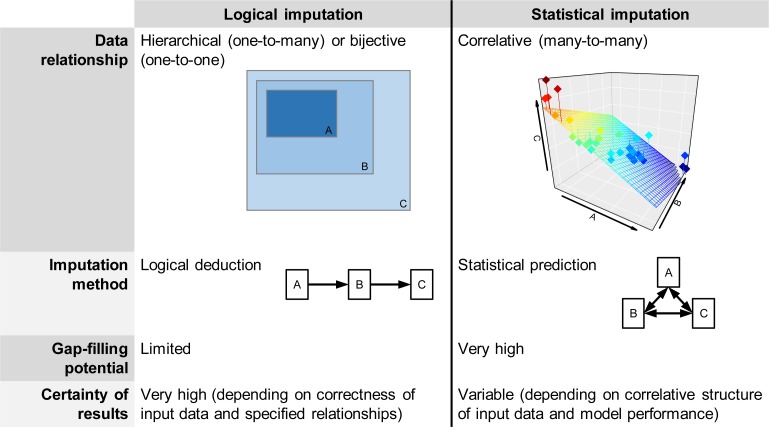
Comparison of logical and statistical data imputation. Logical imputation infers a limited quantity of highly certain data (e.g., deducing woodiness status from growth form), whereas statistical imputation yields large quantities of less certain data (e.g., predicting a suite of functional traits or species occurrences from sparse records).

Data imputation is a technique in which missing or inconsistent data items are replaced with estimated values [51] and represents an inexpensive yet powerful way to improve data coverage in ecological data sets. A conceptual distinction can be made between logical and statistical imputation methods (Fig 2).

Logical imputation uses unequivocal relationships among data to infer new values. This is possible either when data are categorically nested, e.g., trees always being woody [52], or linked by mathematical relationships, e.g., leaf mass per unit area being the inverse of specific leaf area. Although the considerations underlying logical imputation seem rather trivial, many applications in biodiversity data science remain underexploited, e.g., the propagation of information from complex functional traits to more simple ones, the “inheritance” of uniform traits from higher to lower taxonomic groups, or the improvement of regional species checklists based on geographically nested occurrence records or plot data. A major advantage of logical imputation is that the results can be treated with the same certainty as the input data. This makes it a particularly suitable approach for building and extending repositories of primary data. At the same time, logical imputation helps to harmonize data that uses differing terminologies by embedding it in a logical hierarchy (e.g., bee pollination, insect pollination, and animal pollination form nested subsets of pollination syndromes). However, considering that such clear hierarchical relationships are scarce among biodiversity data, the gap-filling potential of logical imputation is limited.

Statistical imputation, on the other hand, utilizes correlative relationships among data to predict new values. Because statistical imputation is based on statistical models, it can incorporate a variety of additional data to refine prediction accuracy. Gap filling techniques for functional traits, e.g., take into account trait–trait, trait–environment, and trait–phylogeny relationships to predict full trait matrices from sparse data [13,53]. Analogously, species distribution models make use of environmental information, species-specific characteristics, or biotic interactions to predict continuous species distributions from point occurrence records [54,55]. Statistical imputation methods allow for the prediction of any number of missing values, but the accuracy of these predictions is always dependent on the quality (i.e., correctness, representativeness, and completeness) of observations and predictor variables as well as the performance of the underlying statistical model. Therefore, statistical imputation is a valuable tool for improving data coverage in specific use cases [10,56,57] but cannot be considered an expansion of primary data.

Strong synergies arise from combining logical imputation, which maximizes the amount of quasiprimary data, with statistical imputation, which may utilize these additional data to improve prediction accuracy. The potential of logical imputation for deducing simple functional traits such as woodiness or growth form is substantial (see case study 1). Although improved knowledge on these traits is of broad ecological interest in itself [58,59], it might be particularly useful to enhance the performance of statistical imputation techniques [13,60]. Similarly, logically imputed distributional information can help to improve species distribution models, e.g., by flagging and removing inconsistent occurrence records [24] or deriving pseudoabsences for species distribution models from regional checklists [61,62].

## Data sharing

Data sharing is a basic condition for data integration [5,63], and although open science initiatives have started to gain traction in ecology, considerable institutional and sociocultural challenges remain [64,65]. Publishers, universities, and funding agencies have a central responsibility for creating an environment in which data sharing is a scientific asset not a disadvantage. Corresponding measures comprise a range of obligations and incentives for data sharing [66,67]. One example for an effective obligation is that many journals now require all data that were used to conduct a study to be stored in open repositories [68]. Likewise, funding agencies strive to improve data quality and long-term accessibility by requiring data management plans [69]. The most important measure, however, is the establishment of adequate incentives for data sharing, primarily by increasing the academic credit gained from doing so. Data citations have been pointed out as a fair and effective way of incentivizing and acknowledging data contributions [67,70] but also alternative measures of research impact and a generally stronger appreciation of data as scientific output will help to open up the ecological research culture [65,67].

## Data integration

Biodiversity data are typically collated and integrated in domain-specific databases that allow fast extraction, exploration, and visualization of normalized data. This approach has transformed the ecological research landscape in the past decades and catalyzed ecological synthesis [4]. However, the scope of any single project is bound to limited technical, financial, and human resources. Building a scalable, dynamic infrastructure to integrate the wealth of existing environmental and ecological data thus requires bundling existing efforts within a unifying framework [32,71].

Distributed networks facilitate the organization of data, resources, and expertise from diverse data holders in a single, collaborative infrastructure that allows for the discovery, acquisition, citation, and (re)use of data [32,72]. A shared data portal acts as a central access point, whereas more specialized databases remain in charge of data aggregation and warehousing [30]. This organizational model has the potential to integrate the heterogeneous ecological data landscape but is also strongly dependent on the broad adoption of data standards. These include, e.g., universal identifiers ranging from standardized species names to digital identifiers for documents, data, and persons (e.g., digital object identifiers [DOIs], Life Science Identifiers (LSIDs), Open Researcher and contributor IDs [ORCIDs]) [73], compatible database structures as well as the implementation of standardized application program interfaces [APIs] and exchange formats [74], rich and well-structured metadata [63,75], and the formalization of existing ecological concepts in ontologies and thesauri [40,76].

The Data Observation Network for Earth (DataONE; https://www.dataone.org, [72]) already provides the basic infrastructure for building an open and distributed network of biodiversity data holders. However, currently the majority of member nodes consists of generic data repositories (e.g., DRYAD) and regional projects (e.g., United States Geological Survey [USGS]), whereas the participation of major aggregators of global plant diversity data (e.g., GBIF) has yet to be realized. Consequently, DataONE currently does not leverage the full potential of its powerful organizational model [63,72]. Some of the future challenges for distributed infrastructures such as DataONE are, e.g., the continuing promotion and development of data standards, the improvement of web-based visualization and analysis capabilities, the incorporation of machine learning for improved data discovery and utilization [77], and the robust implementation of dynamic cross-checking and data imputation workflows for parallel data streams.

## Case studies

We present two case studies based on the GIFT database [28] to demonstrate the importance of data resolution and cross-domain data integration for addressing key questions in macroecology. Considering that GIFT focuses on aggregated data on plant distributions and functional traits only, these case studies provide an outlook on the full potential of an integrated biodiversity data landscape.

### Case study 1: Global patterns in plant growth form

Plant functional types such as growth form capture fundamental axes of ecological variation in a simple way [10,78]. Consequently, knowledge of plant growth form is an important aspect of many ecological applications, ranging from local studies of plant diversity [79] to dynamic global vegetation models [80]. However, despite being a relatively simple and easily determinable trait, data on growth form is still surprisingly scarce and scattered both taxonomically and geographically. Here, we showcase opportunities arising from a systematic integration of aggregated functional and distributional data by predicting growth form spectra across the globe.

We combined angiosperm checklists and growth form data (distinguishing between herb, shrub, and tree) available in GIFT [28]. Oceanic islands as well as geographical units with more than 33% of species lacking growth form information were excluded. From the remaining 818 regional checklists, we included only those species with known growth form status, resulting in a data set containing 1,472,024 species-by-region combinations and 162,300 validated species. We calculated average climatic conditions for all 818 geographical units based on Climatologies at High resolution for the Earth’s Land Surface areas (CHELSA) climate layers [81]. To assess the effects of climate on the relative proportion of growth forms, we used multinomial logistic regression as implemented in the R-package *nnet* [82]. Because our objective was predictive accuracy, not statistical inference, we used all 19 CHELSA bioclimatic variables as predictors without accounting for collinearity [83]. In fitting the model, each observation was weighted by the inverse log-area of the corresponding geographical unit to account for the decreasing representativeness of averaged climatic conditions for larger, climatically more variable regions. We then used the fitted model to predict growth form spectra across a global equal-area hexagon grid (R-package *dggridR* [84]; cell size = 23,300 km^2^).

**Fig 3:**
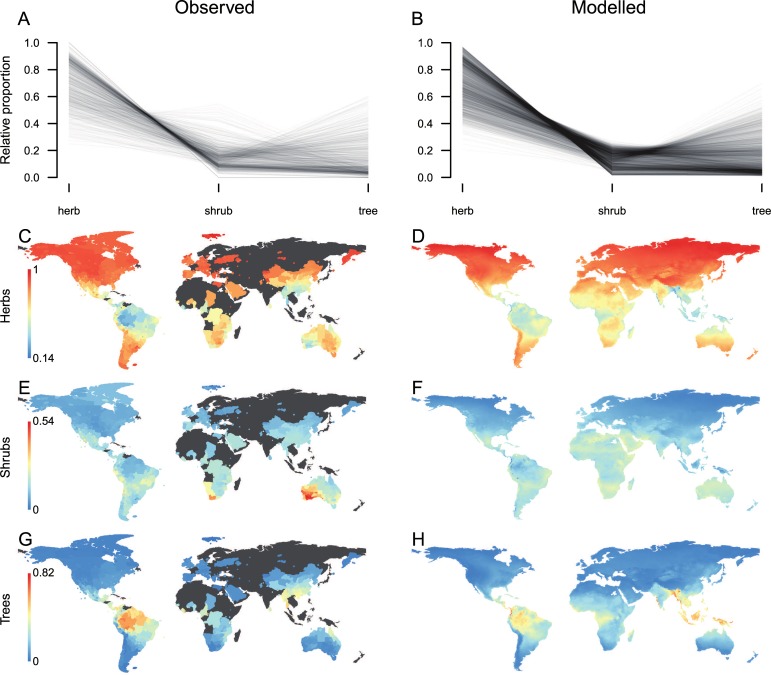
**The global composition in plant growth form as observed for 818 angiosperm floras (left) and modeled for 6,495 equal-area grid cells (right).** Upper plots summarize the growth form spectra across all observed (A) and modeled (B) geographical units, with each line representing a single flora. Lower plots (C–H) show the observed and modeled geographic variation in the proportion of herbs, shrubs, and trees individually. Note that the range of values varies across growth forms. The underlying data and data references for this figure can be found in S1 Data.

Globally, herbs represented the most frequent growth form (Fig 3A and 3C), accounting for 56% of the species and 68% of the species-by-region combinations. Shrubs and trees were less frequent with 23% and 21% of species and 17% and 18% of species-by-region combinations, respectively. Regionally, however, shrubs and trees reached relatively high proportions of species, particularly in Australian scrublands (Fig 3E) and the Amazonian rainforest (Fig 3G). Except for a few local deviations, our predictions of growth form composition were in strong agreement with the observed data (McFadden’s Pseudo-R^2^ = 0.91). Moreover, our results are strongly supported by plot-based analyses of the African and American floras [85,86], which reveal similar geographical trends in growth form composition (S1 Fig and S2 Fig).

In the context of biodiversity data integration, this case study has two implications. First, a near-complete characterization of plant species with respect to fundamental categorical plant traits such as growth form is within reach when exploiting the full potential of data mobilization and imputation. This marks a critical step towards utilizing functional approaches at macroecological scales. Second, aggregated data types reveal remarkably similar but generally smoother biogeographical patterns when compared to comprehensive disaggregated datasets (S1 Fig and S2 Fig). This demonstrates that, due to their high global completeness and representativeness, aggregated data types capture fundamental ecological relationships and produce realistic predictions of plant diversity from regional to global scales.

### Case study 2: The latitudinal gradient in seed mass revisited

Latitude is strongly correlated with numerous environmental characteristics such as temperature, precipitation, seasonality, and long-term climatic stability. Consequently, many aspects of biodiversity show systematic variation along latitude as well [87–89]. Moles and colleagues [90] provide an analysis of the latitudinal variation in seed mass based on a dataset of 11,481 species-by-sites combinations. The authors found a 320-fold decrease in seed mass between the equator and 60 degrees latitude as well as a sudden, 7-fold drop at 23 degrees latitude. These results were linked to changes in vegetation type and growth form composition, leading the authors to posit an abrupt change in plant strategy at the edge of the tropics. Here, our aim is to replicate these findings.

We extracted species lists from GIFT [28] for all mainland units with a complete survey of seed plants. In cases in which geographical units overlapped by more than 5%, we removed the larger unit if floristic data was available at a higher spatial resolution (e.g., preferring federal state- over country-level data); otherwise, we removed the smaller units (e.g., preferring continuous country-level data over patchy national park inventories). Furthermore, we only kept species with information on both seed mass and growth form, yielding a final data set of 519,812 species-by-region combinations and 563 distinct geographical units. In reassessing the relationship between seed mass and latitude, we followed the methodology of Moles and colleagues [90] and used linear regression and piecewise regression.

**Fig 4:**
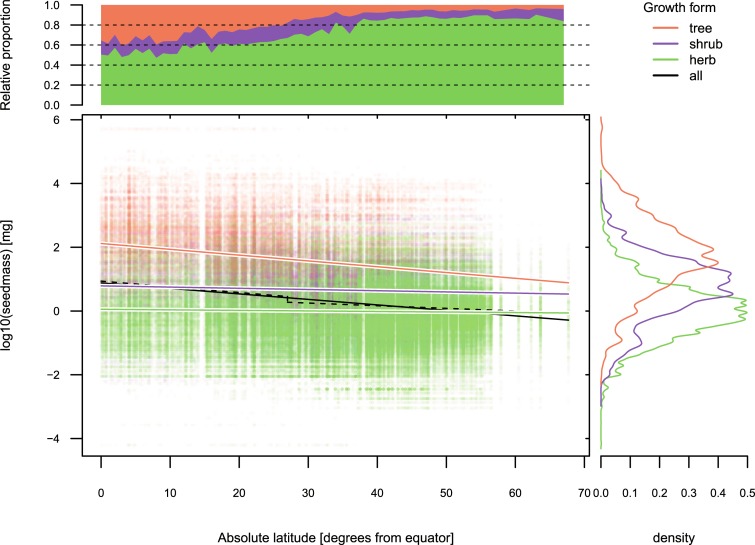
Latitudinal gradient in seed mass for 519,812 species-region combinations. Piecewise regression (dashed black line) was compared against linear models for the entire data set (solid black line) and individual growth forms (colored lines). Upper plot shows the relative proportion of growth forms in each 1-degree latitudinal band. Right-hand plot depicts the frequency distribution of seed mass for individual growth forms. The underlying data and data references for this figure can be found in S2 Data.

We found that the overall decrease in mean seed mass between the equator and 60 degrees latitude was only 11-fold according to linear regression (Fig 4, solid black line) and 8.8-fold according to piecewise regression, the latter indicating a 1.5-fold drop at 27 degrees latitude (Fig 4, dashed black line). Both models had low explanatory power ($R_{linear}^{2}$ = 0.045, $R_{piecewise}^{2}$ = 0.048), reflecting the substantial variation in seed mass at any given latitude. When examining latitudinal variation in seed mass for individual growth forms (Fig 4, colored lines), only trees showed a pronounced decrease towards the poles (12.5-fold), whereas shrubs and herbs exhibited little latitudinal variation (2.1- and 1.3-fold decrease, respectively). In agreement with Moles and colleagues [90], we found a strong latitudinal pattern in the relative proportion of growth forms, with herbs being increasingly dominant at higher latitudes (Fig 4, upper plot). Considering that the logarithmic mean seed mass differs significantly among growth forms (herbs: 0.99 mg, shrubs: 4.59 mg, trees: 48.95 mg; Fig 4, right-hand plot), the overall poleward decrease in seed mass seems to be mostly driven by the replacement of large-seeded trees by small-seeded herbs. According to our data, however, there is no evidence for an abrupt change in plant strategy. In conclusion, we find that the latitudinal gradient in seed mass is considerably less steep than previously reported and lacks a pronounced drop at the edge of the tropics.

This case study illustrates that the quantification of large-scale diversity patterns is highly dependent on the representativeness of the underlying data. In this respect, functional representativeness has been a largely neglected dimension of sample quality. Indeed, the data underlying the original results of Moles and colleagues show remarkably high proportions of tree-dominated biomes and tree species at tropical latitudes (Fig 2B and 2C in [90]). Values of 100% tropical rainforest and 90% tree species at the equator are neither consistent with existing literature [91,92] nor with our data set, which comprises about 50 times more data points (S3 Fig). The representation of trees in Moles and colleagues [90] decreases at the exact point—between 20 and 25 degrees latitude—at which the authors find a sudden drop in seed mass. Thereafter, the latitudinal gradients in growth form composition and seed mass are highly consistent with our data (S3 Fig), suggesting that an uneven latitudinal representation of biomes and growth forms amplified the magnitude and distorted the shape of the latitudinal gradient in seed mass in the study of Moles and colleagues [90]. Integrated biodiversity resources and a targeted utilization of aggregated data types can help to detect and resolve such latent biases to facilitate more robust descriptions of macroecological patterns.

## Conclusion and future directions

The availability, quality, and interoperability of data is paramount to the progress of biogeography and ecology as increasingly data-driven disciplines [5,30,93]. We demonstrate how the explicit consideration of data resolution offers new perspectives on the compilation and integration of plant diversity data. Our results show that a coarse-grained but near-complete knowledge of global plant distributions and basic functional traits is within reach when exploiting the full potential of data mobilization and imputation. This offers exciting opportunities for plant diversity research.

Currently, studies and projects integrating global plant diversity data are mostly based on disaggregated data types. Although this approach has been a successful line of research [10,94,95], the pervasiveness of biases and gaps in disaggregated biodiversity data is of increasing concern to ecologists [14,15,96,97]. We have shown that the systematic utilization of aggregated data can help address this problem (see case studies 1 and 2). First, aggregated data provide a coarse but more complete and less biased picture of geographical variation in taxonomic, functional, and phylogenetic diversity. This offers much-needed baselines against which the completeness of disaggregated data can be evaluated in order to quantify and map gaps in global biodiversity knowledge [16,93]. Second, aggregated data provide prior information about the geographical and statistical distribution of more highly resolved but potentially incomplete or biased ecological variables. This knowledge can be used, e.g., to inform analyses in functional biogeography, to improve species distribution and niche models [98], or to parametrize ancestral state reconstructions [99] and dynamic global vegetation models [100]. Third, aggregated data capitalizes on expert knowledge to compensate for the varying availability and quality of primary (disaggregated) data. Consequently, aggregated data types are not mere compilations of disaggregated data but provide valuable additional information, e.g., reliable species absences or uniform functional traits for higher taxa. These potentials extend to other clades, e.g., mammals, birds, or certain arthropod groups, for which a wealth of literature exists.

Data integration has to bridge not only multiple resolutions but also domains. Satellite-borne, multispectral imagery has become a crucial component of biodiversity research and monitoring, providing global high-resolution data of, e.g., net primary productivity, vegetation cover, or canopy height [101]. Advanced instruments will soon enable the derivation of similar data products for selected functional traits that require integration with in situ observations [102]. Vegetation plot databases are another key source of plant diversity data, holding crucial information on species abundances and interactions. Initiatives like BIEN and sPlot demonstrate how the integration of specimen- and plot data with taxonomic, functional, phylogenetic, and environmental information helps bridge the gap between local-, regional-, and continental-scale ecological processes [8,85,103]. Finally, a better integration of paleontological and socioeconomic data sources with existing biodiversity data resources bears great potential to improve our understanding of biogeography and inform questions concerning conservation planning and alien species management.

The unparalleled pressure on the biosphere renders a full utilization of all available biodiversity data imperative. Rapid advancements in information technology have brought down the technological barriers to this objective. It is now up to ecologists to keep pace with this development and to work collaboratively on creating an integrated biodiversity data landscape that bridges the gap between fine-scale precision and global representativeness.

## Supporting information

S1 Fig **Relative frequency of plant growth forms (herb, shrub, or tree) across the New World derived from disaggregated (left panel, BIEN) versus aggregated (right panel, GIFT) plant diversity data.** (Left) Data from BIEN were obtained through the BIEN r-package by downloading species lists and trait information for 399 geographical units from the New World available in GIFT. The BIEN data set comprised 131,041 species, 969,625 species-by-region combinations, and 69,070 species-by-trait combinations. (Right) The GIFT data set was assembled according to the methodology described in case study 1 and comprised 117,163 species, 940,541 species-by-region combinations, and 89,515 species-by-trait combinations. BIEN, Botanical Information Network and Ecology Network; GIFT, Global Inventory of Floras and Traits.
(DOCX)

S2 Fig **Relative frequency of plant growth forms (herb, shrub, or tree) across central Africa derived from disaggregated plant diversity data (left panel, RAINBIO) versus model predictions derived from aggregated plant diversity data (right panel, GIFT).** (Left) High-resolution plot data from RAINBIO were aggregated to varying spatial resolutions following Watson and colleagues and matched with growth form data available in RAINBIO. (Right) Predictions of growth form composition are based on multinomial logistic regression of a global data set of species checklists and growth form information extracted from the GIFT database (see case study 1 for methodology). GIFT, Global Inventory of Floras and Traits.
(DOCX)

S3 Fig **Data set comparison for case study 2 between Moles and colleagues (2007) (11,481 species-by-sites combinations, upper plot) and GIFT (519,812 species-by-region combinations, lower plot).** GIFT, Global Inventory of Floras and Traits.
(DOCX)

S1 Data Supporting Data for case study 1. (ZIP)

S2 Data Supporting Data for case study 2. (ZIP)

## Abbreviations

API: Application Programming Interface
BIEN: Botanical Information and Ecology Network
CHELSA: Climatologies at High resolution for the Earth’s land Surface Areas
DataONE: Data Observation Network for Earth
DOI: Digital Object Identifier
GBIF: Global Biodiversity Information Facility
GIFT: Global Inventory of Floras and Traits
ILTER: International Long Term Ecological Research network
LSID: Life Science Identifier
ORCID: Open Researcher and Contributor ID
USGS: United States Geological Survey
WCSP: World Checklist of Selected Plant families
