# Supplementary figures and images for: Biodiversity data integration—the significance of data resolution and domain
Source: PLoS Biol. 2019 Mar 18;17(3):e3000183. doi: 10.1371/journal.pbio.3000183 (PMC6445469; doi:10.1371/journal.pbio.3000183)

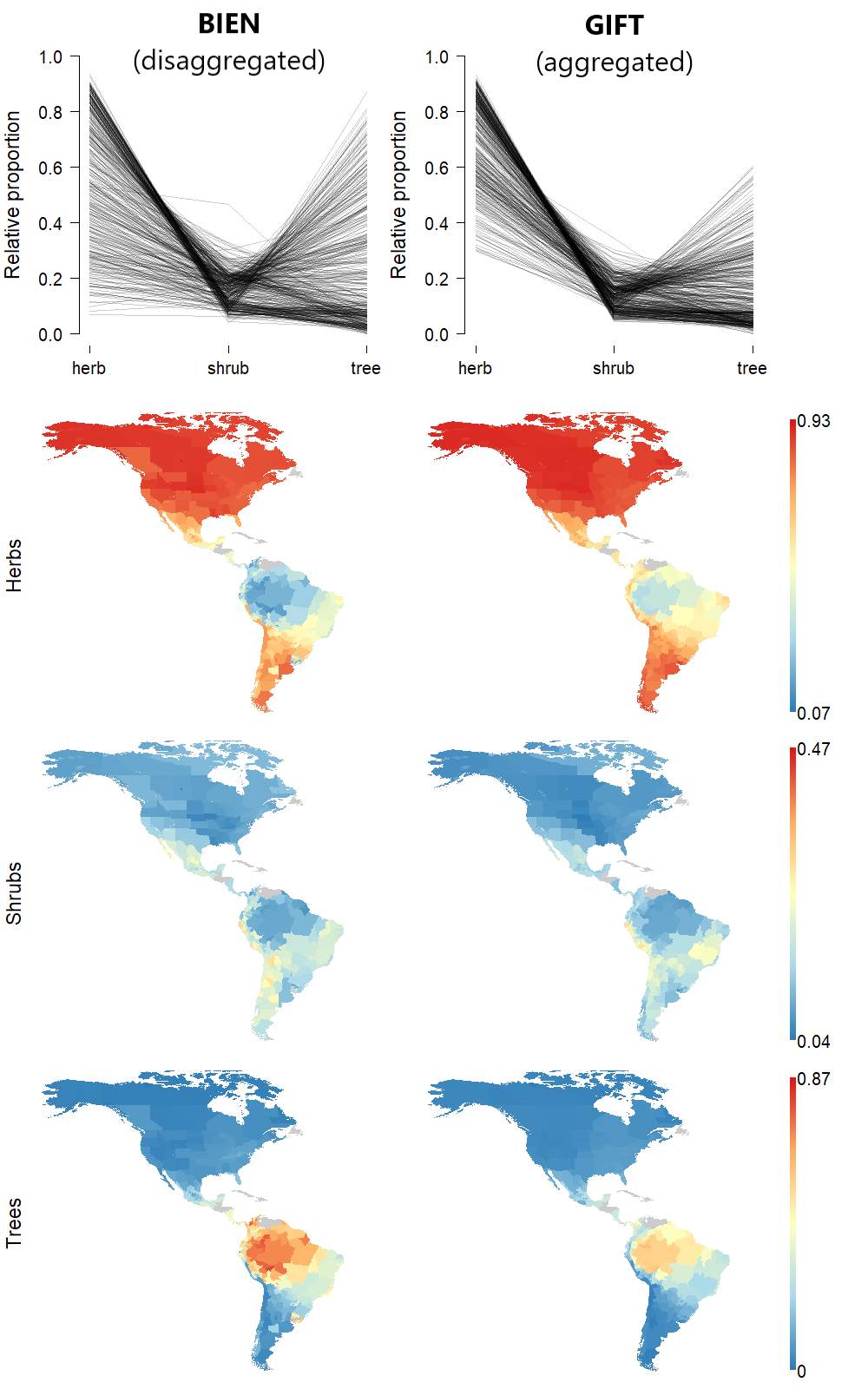

Supplement: S1 Fig — Relative frequency of plant growth forms (herb, shrub, or tree) across the New World derived from disaggregated (left panel, BIEN) versus aggregated (right panel, GIFT) plant diversity data. (Left) Data from BIEN were obtained through the BIEN r-package by downloading species lists and trait information for 399 geographical units from the New World available in GIFT. The BIEN data set comprised 131,041 species, 969,625 species-by-region combinations, and 69,070 species-by-trait combinations. (Right) The GIFT data set was assembled according to the methodology described in case study 1 and comprised 117,163 species, 940,541 species-by-region combinations, and 89,515 species-by-trait combinations. BIEN, Botanical Information Network and Ecology Network; GIFT, Global Inventory of Floras and Traits. (DOCX) [file pbio.3000183.s001.docx]
