# Supplementary material for: Biodiversity data integration—the significance of data resolution and domain
Source: PLoS Biol. 2019 Mar 18;17(3):e3000183. doi: 10.1371/journal.pbio.3000183 (PMC6445469; doi:10.1371/journal.pbio.3000183)
Supplement: S2 Fig — Relative frequency of plant growth forms (herb, shrub, or tree) across central Africa derived from disaggregated plant diversity data (left panel, RAINBIO) versus model predictions derived from aggregated plant diversity data (right panel, GIFT). (Left) High-resolution plot data from RAINBIO were aggregated to varying spatial resolutions following Watson and colleagues and matched with growth form data available in RAINBIO. (Right) Predictions of growth form composition are based on multinomial logistic regression of a global data set of species checklists and growth form information extracted from the GIFT database (see case study 1 for methodology). GIFT, Global Inventory of Floras and Traits. (DOCX) [file pbio.3000183.s002.docx]

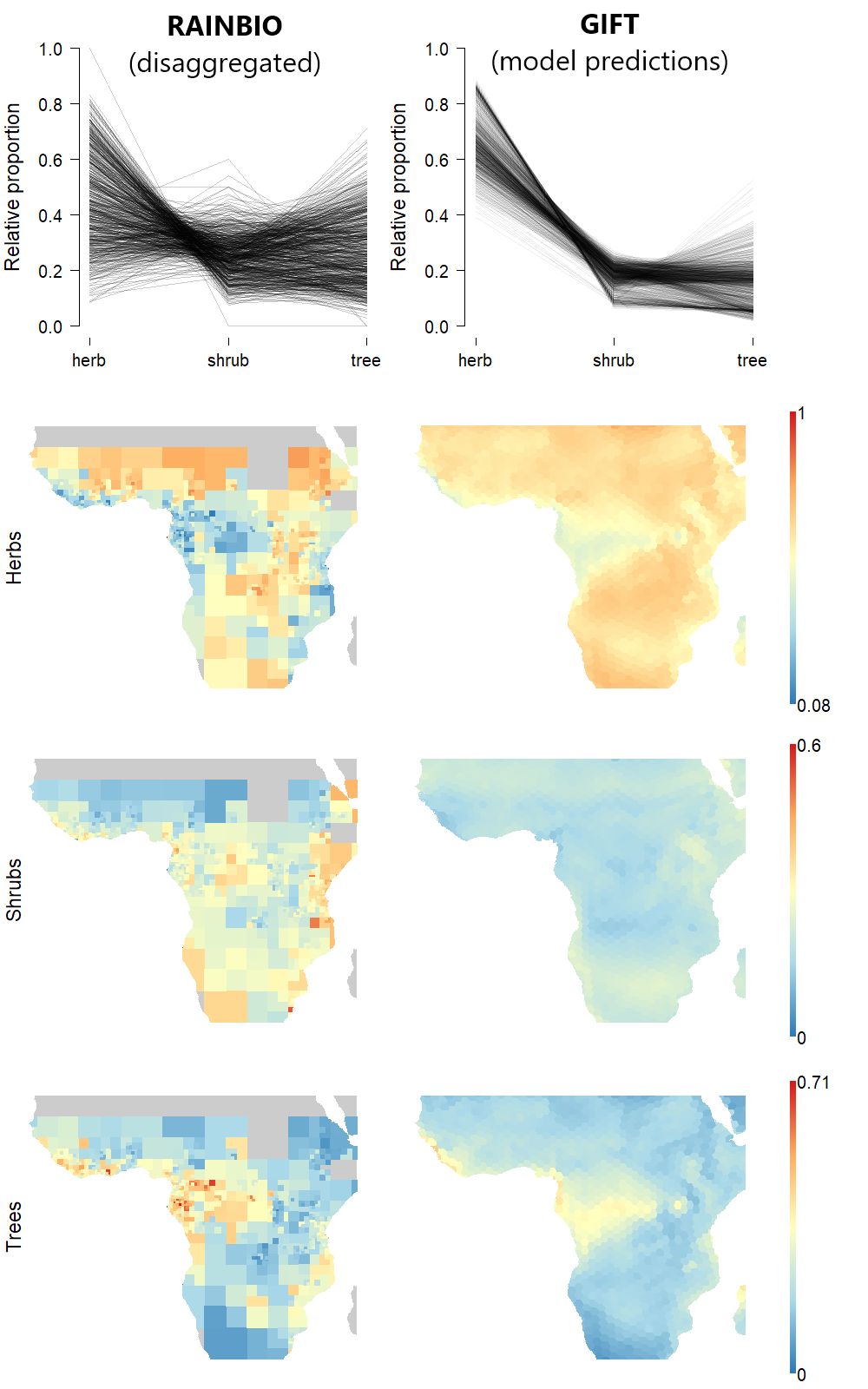


**Reference:**

1. Sosef MSM, Dauby G, Blach-Overgaard A, van der Burgt X, Catarino L, Damen T, et al. Exploring the floristic diversity of tropical Africa. BMC Biology. 2017; 15: 15. doi: 10.1186/s12915-017-0356-8.
