# Supplementary material for: Biodiversity data integration—the significance of data resolution and domain
Source: PLoS Biol. 2019 Mar 18;17(3):e3000183. doi: 10.1371/journal.pbio.3000183 (PMC6445469; doi:10.1371/journal.pbio.3000183)
Supplement: S3 Fig — Data set comparison for case study 2 between Moles and colleagues (2007) (11,481 species-by-sites combinations, upper plot) and GIFT (519,812 species-by-region combinations, lower plot). GIFT, Global Inventory of Floras and Traits. (DOCX) [file pbio.3000183.s003.docx]

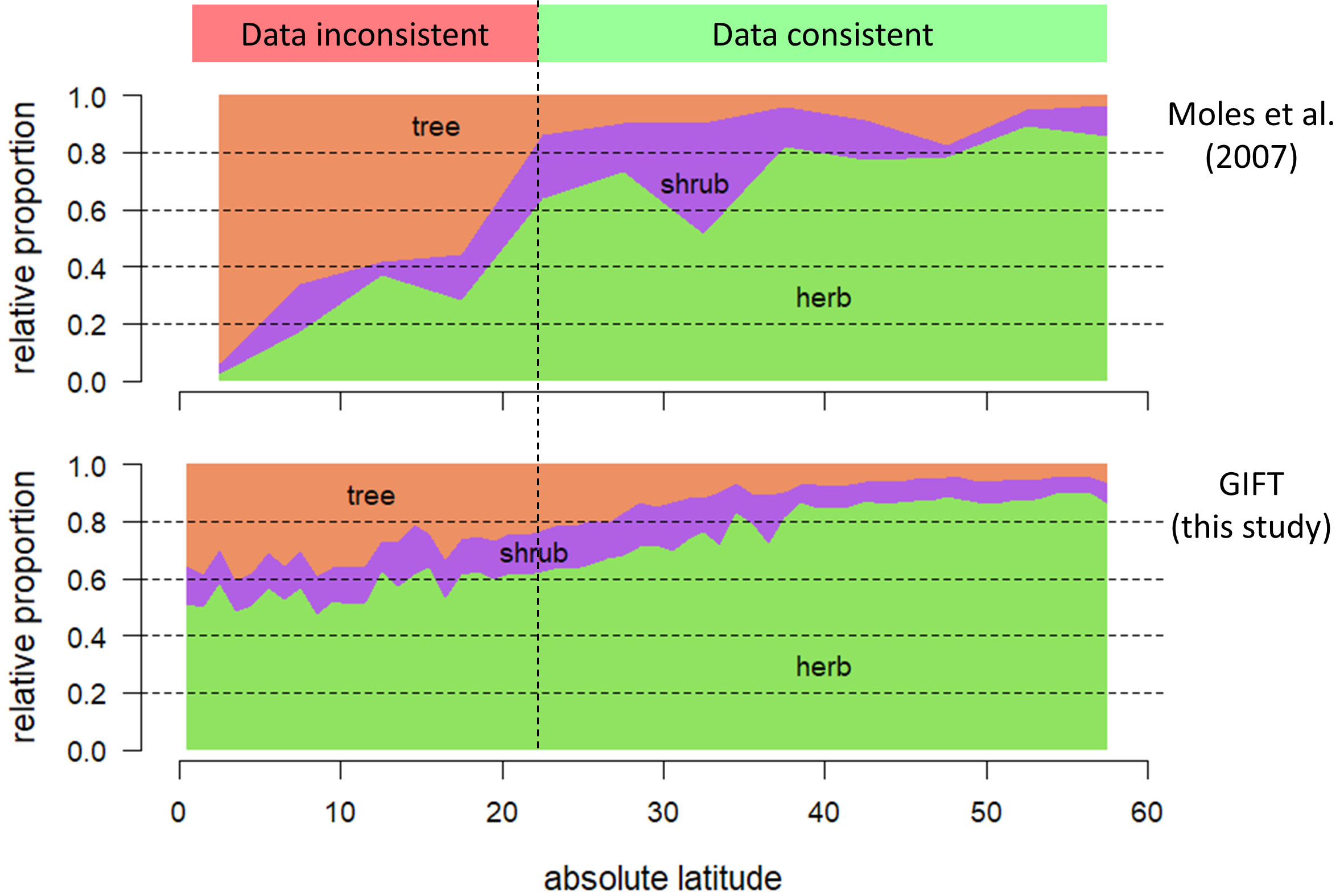


**Reference:**

1. Moles AT, Ackerly DD, Tweddle JC, Dickie JB, Smith R, Leishman MR, et al. Global patterns in seed size. Glob Ecol Biogeogr. 2007; 16: 109–116.
