# Supplementary material for: Biodiversity data integration—the significance of data resolution and domain
Source: PLoS Biol. 2019 Mar 18;17(3):e3000183. doi: 10.1371/journal.pbio.3000183 (PMC6445469; doi:10.1371/journal.pbio.3000183)
Supplement: S1 Data — (ZIP) [file pbio.3000183.s004.zip › S1_references.docx]

| **Reference** | **checklist** | **traits** |
| --- | --- | --- |
| 3D Environmental. Profile for management of the habitats and related ecological and cultural resource values of Boigu Island; 2013 | 0 | 1 |
| Abdel Khalik K, El-Sheikh M, El-Aidarous A. Floristic diversity and vegetation analysis of Wadi Al-Noman, Mecca, Saudi Arabia. Turkish Journal of Botany. 2013; 37: 894–907 | 1 | 1 |
| Abderrahman A, El Kadmiri AA. Richesse et diversité floristique de la subéraie de la Mamora (Maroc). Acta Botanica Malacitana. 2005; 30: 127–138 | 1 | 0 |
| Acevedo-Rodríguez P, Strong MT. Catalogue of the seed plants of the West Indies Website; 2007. Available: http://botany.si.edu/antilles/WestIndies/catalog.htm. Accessed 1 March 2011 | 1 | 0 |
| Adou Yao CY. Diversité floristique et végétation dans le Parc National de Taï, Côte d'Ivoire. Abidjan: Tropenbos Côte d'Ivoire; 2005 | 1 | 0 |
| Alves, Ruy José Válka, Kolbek J. Summit vascular flora of Serra de São José, Minas Gerais, Brazil. Check list. 2009; 5: 35–73 | 1 | 1 |
| Arakaki M, Cano A. Composición florística de la cuenca del río Ilo-Moquegua y Lomas de Ilo, Moquegua, Peru. Revista Peruana de Biología. 2003; 10: 5–19 | 1 | 1 |
| Assédé EPS, Adomou AC, Sinsin B. Magnoliophyta, Biosphere Reserve of Pendjari, Atacora Province, Benin. Check list. 2012; 8: 642–661 | 0 | 1 |
| Azbukina ZM, Bardunov LV, Bezdeleva TA, Bogacheva AV, Bulakh EM, Vasilyeva LN, et al. Flora, vegetation and mycobiota of the reserve «Ussuriysky». Vladivostok: Dalnauka; 2006 | 1 | 0 |
| Badshah L, Hussain F, Sher Z. Floristic inventory, ecological characteristics and biological spectrum of rangeland, District Tank, Pakistan. Pak. J. Bot. 2013; 45: 1159–1168 | 1 | 1 |
| Baker ML, Duretto MF. A census of the vascular plants of Tasmania. Hobart, Australia: Tasmanian Herbarium, Tasmanian Museum and Art Gallery; 2011 | 1 | 0 |
| Bakis Y, Babac MT, Uslu E. Tübives. Turkish Plants Data Service; 2015. Available: http://www.tubives.com/. Accessed 13 February 2015 | 1 | 0 |
| Bancheva S, Vassilev K. Vascular flora of the Beli Lom Nature Reserve in Northeast Bulgaria. Phytologia Balcanica. 2006; 12: 377–386 | 1 | 1 |
| Barker WR, Barker RM, Jessop JP, Vonow HP. Census of South Australian vascular plants. Journal of the Adelaide Botanic Gardens Supplement. 2005; 1: 1–396 | 1 | 0 |
| Belhacene L. Catalogue 2010 des plantes vasculaires du département de la Haute-Garonne. Supplément à Isaatis. 2010; 10: 1–145 | 1 | 0 |
| Benito BM, Lorite J, Pérez-Pérez R, Gómez-Aparicio L, Peñas J, Robertson M. Forecasting plant range collapse in a mediterranean hotspot. When dispersal uncertainties matter. Diversity Distrib. 2014; 20: 72–83. doi: 10.1111/ddi.12148 | 0 | 1 |
| Bernal R, Gradstein SR, Celis M. Catálogo de plantas y líquenes de Colombia; 2015. Available: http://catalogoplantasdecolombia.unal.edu.co/. Accessed 15 January 2016 | 1 | 1 |
| BGCI. GlobalTreeSearch online database; 2017. Available: www.bgci.org/globaltree_search.php. Accessed 14 August 2017 | 0 | 1 |
| Bingham MG, Willemen A, Wursten BT, Ballings P, Hyde MA. Flora of Zambia; 2016. Available: http://www.zambiaflora.com/. Accessed 14 November 2016 | 1 | 0 |
| BioScripts. Flora Vascular; 2014. Available: http://www.floravascular.com/. Accessed 25 May 2014 | 1 | 1 |
| Botanical Garden Tel Aviv. Israel Flora: Tel Aviv University | 1 | 1 |
| Bowdoin Scientific Station. Vascular plants of Kent Island; 2011. Available: https://www.bowdoin.edu/kent-island/species/plants.shtml. Accessed 14 September 2011 | 1 | 0 |
| Brennan K. An annotated checklist of the vascular plants of the Alligator Rivers Region, Northern Territory, Australia. Barton, Australia: Supervising Scientist; 1996 | 1 | 1 |
| Bridgewater SGM, Harris DJ, Whitefoord C, Monro AK, Penn MG, Sutton DA, et al. A Preliminary Checklist of the vascular plants of the Chiquibul Forest, Belize. Edin. Jnl of Bot. 2006; 63: 269–321 | 1 | 1 |
| Broughton DA, McAdam JH. A checklist of the native vascular flora of the Falkland Islands (Islas Malvinas). new information on the species present, their ecology, status and distribution. The Journal of the Torrey Botanical Society. 2005; 132: 115–148 | 1 | 0 |
| Brundu G, Camarda I. The Flora of Chad: a checklist and brief analysis. Phytokeys. 2013; 23: 1–17 | 1 | 1 |
| Buchwald E, Wind P, Bruun HH, Møller PF, Ejrnæs R, Svart HE. Hvilke planter er hjemmehørende i Danmark. Jydsk Naturhistorisk Forening. 2013; 118: 72–96 | 1 | 0 |
| Bundesamt für Naturschutz. Floraweb; 2016. Available: www.floraweb.de. Accessed 15 September 2016 | 1 | 1 |
| Bundesamt für Naturschutz. Floraweb; 2016. Available: www.floraweb.de. Accessed 15 September 2016 | 1 | 1 |
| Butler BJ, Barclay JS, Fisher JP. Plant communities and flora of Robins Island (Long Island), New York. Journal of the Torrey Botanical Society. 1999; 126: 63–76 | 1 | 0 |
| CARMABI. Dutch Caribbean Biodiversity Explorer; 2009. Available: http://www.dcbiodata.net/explorer/home. Accessed 24 June 2011 | 1 | 0 |
| Cascante-Marín A, Estrada-Chavarría A. Las plantas vasculares de El Rodeo, Costa Rica. Brenesia. 2012; 77: 71–128 | 1 | 1 |
| Case TJ, Cody ML, Ezcurra E. A new island biogeography of the Sea of Cortés. New York, NY: Oxford University Press; 2002 | 1 | 0 |
| Catarino L, Martins ES, Basto MF, Diniz MA. An annotated checklist of the vascular flora of Guinea-Bissau (West Africa). Blumea-Biodiversity, Evolution and Biogeography of Plants. 2008; 53: 1–222 | 1 | 1 |
| Chang C-S, Kim H, Chang K. Provisional Checklist of the Vascular Plants for the Korea Peninsular Flora (KPF). Version 1.0. Korea | 1 | 0 |
| Chang C-S, Kim H, Chang K. Provisional Checklist of the Vascular Plants for the Korea Peninsular Flora (KPF). Version 1.0. Korea | 1 | 0 |
| Chawla A, Parkash O, Sharma V, Rajkumar S, Lal B, Gopichand, et al. Vascular plants, Kinnaur, Himachal Pradesh, India. Check list. 2012; 8: 321–348 | 1 | 1 |
| Cheffings CM, Farrell L, editors. The vascular plant red data list for Great Britain. Peterborough: Joint Nature Conservation Committee; 2005 | 1 | 0 |
| Chiapella J, Ezcurra C. La flora del parque provincial Tromen, provincia de Neuquén, Argentina. Multequina. 1999; 8: 51–60 | 1 | 0 |
| Chinese Virtual Herbarium. The Flora of China v. 5.0; 2016. Available: http://www.cvh.org.cn/. Accessed 15 January 2016 | 1 | 1 |
| Chong KY, Tan TWH, Corlett RT. A checklist of the total vascular plant flora of Singapore. Native, Naturalised and Cultivated Species. Singapore: Raffles Museum of Biodiversity Research; 2009 | 1 | 1 |
| Christodoulakis D. The flora of Ikaria (Greece, E. Aegean Islands). Phyton. 1996; 36: 63–91 | 1 | 0 |
| Clark JL, Neill DA, Asanza M. Floristic checklist of the Mache-Chindul mountains of Northwestern Ecuador. Contributions from the United States National Herbarium. 2006; 54: 1–180 | 1 | 1 |
| Cochard R, Bloesch U. Electronic plant species database of the Saadani National Park, coastal Tanzania; 2007. Available: http://www.wildlife-baldus.com/saadani.html. Accessed 14 November 2016 | 1 | 1 |
| CONABIO. Sistema Nacional de Información sobre Biodiversidad; 2016. Available: https://www.gob.mx/conabio. Accessed 11 April 2016 | 1 | 0 |
| Conti F, Abbate G, Alessandrini A, Blasi C. Annotated Checklist of the Italian Vascular Flora. Italy: Palombi Editori, Roma; 2005 | 1 | 0 |
| Conti F, Bartolucci F. The Vascular Flora of the National Park of Abruzzo, Lazio and Molise (Central Italy). Cham: Springer International Publishing; 2015 | 1 | 0 |
| Costion C, Lorence D. The endemic plants of Micronesia. A geographical checklist and commentary. Micronesica. 2012; 43: 51–100 | 0 | 1 |
| Cuc Phuong National Park. Flora of the Cuc Phuong National Park. Vietnam; 2011 | 1 | 0 |
| Da Vela M, Frignani F, Bonari G, Angiolini C. La flora vascolare della diserva naturale "La Pietra" (Toscana meridionale). Micol. Veget. Medit. 2013; 28: 135–160 | 1 | 1 |
| Danihelka J, Chrtek J, Kaplan Z. Checklist of vascular plants of the Czech Republic. Preslia. 2012; 84: 647–811 | 1 | 0 |
| D'Arcy WG. The island of Anegada and its flora. Atoll Research Bulletin. 1971; 139: 1–21 | 1 | 0 |
| Dauby G, Leal M, Stevart T. Vascular plant checklist of the coastal National Park of Pongara, Gabon. Systematics and geography of plants. 2008; 78: 155–216 | 1 | 1 |
| Dauby G, Zaiss R, Blach-Overgaard A, Catarino L, Damen T, Deblauwe V, et al. RAINBIO. A mega-database of tropical African vascular plants distributions. PK. 2016; 74: 1–18. doi: 10.3897/phytokeys.74.9723 | 1 | 1 |
| de la Luz, León, Rebman J, Domínguez-León M, Domínguez-Cadena R. The vascular flora and floristic relationships of the sierra de la Giganta in Baja California Sur, México. Revista mexicana de biodiversidad. 2008; 79: 29–65 | 1 | 1 |
| de Medeiros, Marcelo Brilhante, Walter BMT, Da Silva, Glocimar Pereira, Gomes BM, Lima, Isabela Lustz Portela, Silva SR, et al. Vascular flora of the Tocantins river middle basin, Brazil. Check list. 2012; 8: 852–885 | 1 | 0 |
| de V. Barbosa, Maria Regina, Thomas WMW, Zárate ELdP, de Lima RB, de Fátima Agra M, de Lima IB, et al. Checklist of the vascular plants of the Guaribas Biological Reserve, Paraíba, Brazil. Revista Nordestina de Biologia. 2011; 20: 79–106 | 1 | 0 |
| Desmet P, Brouillet L. Database of Vascular Plants of Canada (VASCAN): a community contributed taxonomic checklist of all vascular plants of Canada, Saint Pierre and Miquelon, and Greenland. Phytokeys. 2013; 25: 55–67 | 0 | 1 |
| Dimopoulos P, Raus T, Bergmeier E, Constantinidis T, Iatrou G, Kokkini S, et al. Vascular plants of Greece. An annotated checklist. Berlin, Athens: Botanischer Garten und Botanisches Museum Berlin-Dahlem, Freie Universität Berlin; Hellenic Botanical Society; 2013 | 1 | 1 |
| Domínguez E, Marticorena C, Elvebakk A, Pauchard A. Catálogo de la flora vascular del Parque Nacional Pali Aike. XII Región, Chile. Gayana Botánica. 2004; 61: 67–72 | 1 | 1 |
| Doroftei M, Oprea A, Ştefan N, Sârbu I. Vascular wild flora of Danube Delta Biosphere Reserve. Sci. Annals of Danube Delta Institute. 2011; 17: 15–52 | 1 | 0 |
| Dowhan JJ, Rozsa R. Flora of Fire Island, Suffolk County, New York. Bulletin of the Torrey Botanical Club. 1989; 116: 265–282 | 1 | 0 |
| Drummond RB, Mapaure I. List of Flowering Plants and Ferns (of Chirinda Forest); 1994. Available: http://www.zimbabweflora.co.zw/speciesdata/checklist-display.php?checklist_code=4. Accessed 20 January 2015 | 1 | 0 |
| Egea J de, Peña-Chocarro M, Espada C, Knapp S. Checklist of vascular plants of the Department of Ñeembucú, Paraguay. Phytokeys. 2012; 9: 15–179 | 1 | 1 |
| Ejaz-ul-Islam Dar M, Cochard R, Shrestha RP, Ahmad S. Floristic composition of Machiara National Park, District Muzaffarabad Azad Kashmir, Pakistan. International Journal of Biosciences. 2012; 2: 28–45 | 1 | 1 |
| Eleftheriadou E, Raus T. The vascular flora of the nature reserve Frakto Virgin Forest of Nomos Dramas (E Makedonia, Greece). Willdenowia. 1996; 25: 455–485 | 1 | 0 |
| El-Ghani MM, Abdel-Khalik KN. Floristic Diversity and Phytogeography of the Gebel Elba National Park, South-East Egypt. Turkish Journal of Botany. 2005; 30: 121–136 | 1 | 1 |
| Engemann K, Sandel B, Boyle BL, Enquist BJ, Jørgensen PM, Kattge J, et al. A plant growth form dataset for the New World. Ecology. 2016; 97: 3243. doi: 10.1002/ecy.1569 | 0 | 1 |
| Espinosa-Jiménez JA, Pérez-Farrera MÁ, Martínez-Camilo R. Inventario florístico del Parque Nacional Cañón del Sumidero, Chiapas, México. Boletín de la Sociedad Botánica de México. 2011; 89: 37–82 | 1 | 1 |
| Evarts-Bunders P, Evarte-Bundere G, Bāra J, Nitcis M. The flora of vascular plants in nature reserve „Eglone”. Acta Biologica Universitatis Daugavpiliensis. 2013; 13: 21–38 | 1 | 0 |
| Fern K, Fern A. Useful Tropical Plants Database; 2015. Accessed 25 July 2015 | 0 | 1 |
| Ferreira AL, Coutinho BR, Pinheiro HT, Thomaz LD. Composição florística e formações vegetais da Ilha dos Franceses, Espírito Santo. Bol. Mus. Biol. Mello Leitao. 2007; 22: 25–44 | 0 | 1 |
| Figueiredo E, Paiva J, Stevart T, Oliveira F, Smith GF. Annotated catalogue of the flowering plants of São Tomé and Príncipe. Bothalia. 2011; 41: 41–82 | 0 | 1 |
| Figueroa-C. Y, Galeano G. Lista comentada de las plantas vasculares del enclave seco interandino de La Tatacoa (Huila, Colombia). Caldasia. 2007; 29: 263–281 | 1 | 1 |
| Fischer E, Rembold K, Althof A, Obholzer J, Malombe I, Mwachala G, et al. Annotated checklist of the vascular plants of Kakamega Forest, Western Province, Kenya. Journal of East African Natural History. 2010; 99: 129–226 | 1 | 1 |
| Fischer MA, Adler W, Oswald K. Exkursionsflora für Österreich, Liechtenstein und Südtirol. Bestimmungsbuch für alle in der Republik Österreich, im Fürstentum Liechtenstein und in der Autonomen Provinz Bozen. 3rd ed. Linz: OÖ Landesmuseum; 2008 | 1 | 1 |
| Funk VA, Hollowell T, Berry P, Kelloff C, Alexander SN. Checklist of the plants of the Guiana Shield (Venezuela: Amazonas, Bolivar, Delta Amacuro; Guyana, Surinam, French Guiana). Washington, DC: Department of Botany, National Museum of Natural History; 2007 | 1 | 0 |
| Gafurova ММ. Vascular plants of Chuvash Republic. Flora of the Volga River Basin V. III: The Russian Academy of Sciences; 2014 | 1 | 0 |
| Gage S, Joneson SL, Barkalov VY, Eremenko NA, Takahashi H. A newly compiled checklist of the vascular plants of the Habomais, the Little Kurils. Bulletin of the Hokkaido University Museum. 2006; 3: 67–91 | 1 | 0 |
| Gamit SB, Maurya RR, Qureshimatva UM, Solanki HA. Check list of flowering plants in Tapi District, Gujarat, India. International Journal of advanced Research. 2015; 3: 1104–1123 | 0 | 1 |
| Gnoumou A, Ouedraogo O, Schmidt M, Thiombiano A. Floristic diversity of classified forest and partial faunal reserve of Comoé-Léraba, southwest Burkina Faso. Check list. 2015; 11: 1557 | 1 | 1 |
| Grozeva NH. The flora of Atanasovsko lake natural reserve. In: Gruev B, Nikolova M, Donev A, editors. Proceedings of the Balkan Scientific Conference of Biology; 2005. pp. 381–396 | 1 | 0 |
| Harris DJ. The vascular plants of the Dzanga-Sangha Reserve, Central African Republic. Edinburgh, Scotland, UK: Royal Botanic Garden Edinburgh; 2002 | 1 | 1 |
| Hauenstein E, Muñoz-Pedreros A, Yánez J, Sánchez P, Möller P, Guiñez B, et al. Flora y vegetación de la Reserva Nacional Lago Peñuelas, Reserva de la Biósfera, Región de Valparaíso, Chile. Bosque (Valdivia). 2009; 30: 159–179. doi: 10.4067/S0717-92002009000300006 | 1 | 1 |
| Hawkins BA, Rueda M, Rangel TF, Field R, Diniz-Filho JAF, Linder P. Community phylogenetics at the biogeographical scale: cold tolerance, niche conservatism and the structure of North American forests. J. Biogeogr. 2013; 41: 23–38 | 0 | 1 |
| Heberling JM, Jo I, Kozhevnikov A, Lee H, Fridley JD. Biotic interchange in the Anthropocene. Strong asymmetry in East Asian and eastern North American plant invasions. Global Ecol. Biogeogr. 2017; 26: 447–458. doi: 10.1111/geb.12551 | 0 | 1 |
| Hill SR. An annotated checklist of the vascular flora of Assateague Island (Maryland and Virginia). Castanea. 1986; 51: 265–305 | 1 | 0 |
| Hoang VS. Uses and conservation of plant diversity in Ben En National Park, Vietnam. Ph.D., National Herbarium of the Netherlands, Leiden University Branch. 2009 | 1 | 1 |
| Hoke P, Demey R, Peal A. A Rapid Biological Assessment of North Lorma, Gola and Grebo National Forests, Liberia. Arlington, USA: Conservation International; 2007 | 1 | 0 |
| Hyde MA, Wursten BT, Ballings P, Coates Palgrave M. Flora of Botswana; 2016. Available: http://www.botswanaflora.com/. Accessed 14 November 2016 | 1 | 0 |
| Hyde MA, Wursten BT, Ballings P, Coates Palgrave M. Flora of Malawi; 2016. Available: http://www.malawiflora.com/index.php. Accessed 14 November 2016 | 1 | 0 |
| Hyde MA, Wursten BT, Ballings P, Coates Palgrave M. Flora of Mozambique; 2016. Available: http://www.mozambiqueflora.com/. Accessed 14 November 2016 | 1 | 0 |
| Hyde MA, Wursten BT, Ballings P, Coates Palgrave M. Flora of Zimbabwe; 2016. Accessed 14 November 2016 | 1 | 0 |
| ICIMOD. Hindu Kush-Himalayan (HKH) Conservation Portal; 2015. Available: http://www.icimod.org/hkhconservationportal/Initiatives.aspx. Accessed 21 April 2015 | 1 | 0 |
| INBIO. Lista de planta de Costa Rica. With updates by Eduardo Chacón; 2000. Available: http://www.inbio.ac.cr/papers/manual_plantas/index.html. Accessed 5 February 2016 | 1 | 0 |
| Jackes BR. Plants of Magnetic Island. 3rd ed. Townsville, Australia: James Cook University; 2010 | 1 | 0 |
| Jardim Botânico do Rio de Janeiro. Flora do Brasil 2020 em construção; 2016. Available: http://floradobrasil.jbrj.gov.br/. Accessed 9 May 2016 | 1 | 1 |
| Jasprica N, Dolina K, Milović M. Plant taxa and communities on three islets in south Croatia, NE Mediterranean. Nat Croat. 2015; 24: 191–213. doi: 10.20302/NC.2015.24.12 | 0 | 1 |
| Jiménez JE, Juárez P, Díaz A. Checklist of the vascular flora of Reserva Biológica San Luis, Costa Rica. Check list. 2016; 12: 1859. doi: 10.15560/12.2.1859 | 1 | 1 |
| Jordano P. FRUBASE; 2008. Available: http://ebd10.ebd.csic.es/mywork/frubase/frubase.html. Accessed 26.06.15 | 0 | 1 |
| Kabuye CHS, Mungai GM, Mutangah JG. Flora of Kora National Reserve. In: Coe M, Collins NM, editors. Kora: An Ecological Inventory of the Kora National Reserve, Kenya. Kora Research Project 1982-85 : a Joint Venture Between the National Museums of Kenya and the Royal Geographical Society: Royal Geographical Society; 1986. pp. 57–104 | 1 | 1 |
| Kamari G, Phitos D, Snogerup B, Snogerup S. Flora and vegetation of Yioura, N Sporades, Greece. Willdenowia. 1988; 17: 59–85 | 1 | 0 |
| Karlsson T. Lista över Östergötlands kärlväxter; 2011. Available: http://ostgotaflora.se/handledning.html. Accessed 17 May 2016 | 1 | 0 |
| Kattge J, Diaz S, Lavorel S, Prentice IC, Leadley P, Bönisch G, et al. TRY – a global database of plant traits. Global Change Biology. 2011; 17: 2905–2935 | 0 | 1 |
| Kattge J, Diaz S, Lavorel S, Prentice IC, Leadley P, Bönisch G, et al. TRY – a global database of plant traits. Global Change Biology. 2011; 17: 2905–2935 | 0 | 1 |
| Kelloff CL, Funk VA. Preliminary checklist of the plants of Kaieteur National Park, Guyana. Washington: National Museum of Natural History, Smithsonian Institution; 1998 | 1 | 0 |
| Kelly L. The vascular flora of Huggins Island, Onslow County, North Carolina. Castanea. 2006; 71: 295–311 | 1 | 0 |
| Kemenes A. Distribuição espacial da flora terrestre fanerogâmica do Parque Nacional Marinho de Abrolhos, BA. Revista Brasil. Bot. 2003; 26: 141–150 | 0 | 1 |
| Kenneally KF. Ashmore Reef and Cartier Island. Species lists; 1993. Available: http://www.environment.gov.au/biodiversity/abrs/online-resources/flora/50/index.html. Accessed 6 April 2011 | 1 | 0 |
| Keppel G. Summary report on forests of the Mataqali Nadicake Kilaka, Kubulau district, Bua, Vanua Levu; 2005 | 0 | 1 |
| Khanina L, Zaugolnova L, Smirnova O, Shovkun M, Glukhova E. Flora of vascular plants in the Central European Russia; 2016. Available: http://www.impb.ru/eco/index.php?l=en. Accessed 6 September 2017 | 1 | 0 |
| Kim C-S, Koh J-G, Moon M-O, Song G-P, Hyun H-J, Song K-M, et al. Flora and life form spectrum of Hallasan natural reserve, Korea. Journal of the Environmental Sciences. 2007; 16: 1257–1269 | 0 | 1 |
| Kim H-J, Ji S-J, Jung S-Y, Park SH, Lee S-G, Lee C-W, et al. Flora of Vascular Plants in Deokjeokdo (Ongjin-gun) and Its Adjacent Regions, Korea. Korean Journal of Plant Resources. 2015; 28: 487–510. doi: 10.7732/kjpr.2015.28.4.487 | 1 | 0 |
| Kleyer M, Bekker RM, Knevel IC, Bakker JP, Thompson K, Sonnenschein M, et al. The LEDA Traitbase: a database of life-history traits of the Northwest European flora. J Ecol. 2008; 96: 1266–1274 | 0 | 1 |
| Klimeš L, Dickoré B. Flora of Ladak (NW Himalaya). A preliminary check-list; 2015. Available: http://www.butbn.cas.cz/klimes/desert.html. Accessed 12 January 2015 | 1 | 0 |
| Koltzenberg M. Flora von Kamtschatka. Vorläufige Checkliste; 2002 | 1 | 0 |
| Koltzenburg M. Checkliste der Gefässpflanzen Irlands; 2011. Available: http://www.saxifraga.de/eire/irl_artenlisten_gp.html. Accessed 4 April 2011 | 1 | 0 |
| Kraaij T. The flora of the Bontebok National Park in regional perspective. South African Journal of Botany. 2011; 77: 455–473. doi: 10.1016/j.sajb.2010.09.013 | 1 | 1 |
| Kraft TS, Wright SJ, Turner I, Lucas PW, Oufiero CE, Supardi Noor MN, et al. Seed size and the evolution of leaf defences. J Ecol. 2015; 103: 1057–1068. doi: 10.1111/1365-2745.12407 | 0 | 1 |
| Kumar A, Bajpai O, Mishra AK, Sahu N, Behera SK, Bargali SS, et al. A checklist of the flowering plants of Katerniaghat Wildlife Sanctuary, Uttar Pradesh, India. J. Threat. Taxa. 2015; 7: 7309–7408 | 0 | 1 |
| Kuzmenkova SM. Plants of Belarus; 2015. Available: http://hbc.bas-net.by/plantae/eng/default.php. Accessed 15 February 2016 | 1 | 0 |
| Laliga LS, Benavent, JEO, Conca A, Signes JXS, Nebot JR. Catálogo de la flora del Parque Natural de la Sierra de Mariola (Alicante-Valencia). Flora Montiberica. 2012; 51: 97–125 | 1 | 0 |
| Lazkov GA, Sultanova BA. Checklist of vascular plants of Kyrgyzstan. Helsinki: Botanical Museum, Finnish Museum of Natural History; 2011 | 1 | 0 |
| Le Houerou HN. Plant diversity in Marmarica (Libya & Egypt): a catalogue of the vascular plants reported with their biology, distribution, frequency, usage, economic potential, habitat and main ecological features, with an extensive bibliography. Candollea. 2004; 59: 259–308 | 1 | 1 |
| Lee R-Y, Jang H-D, Kim Y-Y, Yang S-G, Choi H-J, Ji S-J, et al. Flora of vascular plants in the Chilgapsan Provincial Park, Korea. Journal of Asia-Pacific Biodiversity. 2014; 7: 237–247 | 1 | 0 |
| Lee S-M, Lee H-Y, Lee Y-M, Park S-H, Lee B-C, Lim W-H. Flora of Gyeongju National Park, Korea. Journal of Korean Nature. 2008; 1: 21–38 | 1 | 0 |
| Lester-Garland LV. A flora of the islands of Jersey. with a list of the plants of the Channel Islands in general, and remarks upon their distribution and geographical affinities. London, UK: West, Newman & Co; 1903 | 1 | 0 |
| Limbu D, Koirala M, Shang Z. A Checklist of Angiospermic Flora of Tinjure-Milke-Jaljale, Eastern Nepal. Nepal Journal of Science and Technology. 2013; 13: 87–96 | 0 | 1 |
| Linhart YB. Local biogeography of plants on a Caribbean atoll. Journal of Biogeography. 1980; 7: 159–171 | 0 | 1 |
| Lipkin R. Aniakchak National Monument and Preserve, vascular plant inventory: final technical report. Anchorage, USA: National Park Service, Southwest Alaska Network Inventory & Monitroing Program; 2005 | 1 | 0 |
| Lopez-Martinez JO, Sanaphre-Villanueva L, Dupuy JM, Hernandez-Stefanoni JL, Meave JA, Gallardo-Cruz JA. Beta-Diversity of functional groups of woody plants in a tropical dry forest in Yucatan. PLoS One. 2013; 8: e73660. doi: 10.1371/journal.pone.0073660 | 0 | 1 |
| Lorite J. An updated checklist of the vascular flora of Sierra Nevade (SE Spain). Phytotaxa. 2016; 261: 1–57 | 1 | 0 |
| Luke Q. Annotated Checklist of the Plants of the Shimba Hills, Kwale District, Kenya. Journal of East African Natural History. 2005; 94: 5–120 | 1 | 1 |
| Luna-Jorquera G, Fernández CE, Rivadeneira MM. Determinants of the diversity of plants, birds and mammals of coastal islands of the Humboldt current systems. Implications for conservation. Biodivers Conserv. 2012; 21: 13–32. doi: 10.1007/s10531-011-0157-2 | 1 | 0 |
| Marquand ED. Flora of Guernsey and the lesser Channel Islands. namely Alderney, Sark, Herm, Jethou, and the adjacent islets. London, UK: Dulau & Co; 1901 | 1 | 0 |
| Marticorena C, Squeo FA, Arancio G, Muñoz M. Catálogo de la flora vascular de la IV Región de Coquimbo. In: Squeo FA, Arancio G, Gutiérrez JR, editors. Libro rojo de la flora nativa y de los sitios prioritarios para su conservación: Región de Atacama: Ediciones Universidad de La Serena La Serena; 2008. pp. 105–142 | 1 | 1 |
| Marticorena C, Stuessy TF, Baeza CM. Catalogue of the vascular flora of the Robinson Crusoe or Juan Fernández islands, Chile. Gayana Botánica. 1998; 55: 187–211 | 0 | 1 |
| Masharabu T. Flore et végétation du Parc National de la Ruvubu au Burundi: diversité, structure et implications pour la conservation | 1 | 1 |
| McCrea J. Inventory of the land conservation values of the Houtman Abrolhos Islands. Perth, Australia: Department of Fisheries, Government of Western Australia; 2003 | 1 | 0 |
| Medina R, Reina-E M, Herrera E, Ávila FA, Chaparro O, Cortés-B. R. Catálogo preliminar da flora vascular dos bosques subandinos da cuchilla El Fara (Santander-Colômbia). Colombia Forestal. 2010; 13: 55–85 | 1 | 1 |
| Medjahdi B, Ibn Tattou M, Barkat D, Benabedli K. La flore vasculaire des Monts des Trara (Nord Ouest Algérien). Acta Botanica Malacitana. 2009; 34: 57–75 | 1 | 1 |
| Memariani F, Joharchi MR, Ejtehadi H, Emadzadeh K. Contributions to the flora and vegetation of Binalood Mountain range, NE Iran: Floristic and chorological studies in Fereizi region. Ferdowsi University International Journal of Biological Sciences. 2009; 1: 1–17 | 1 | 1 |
| Ministry of Environment. The national red list 2012 of Sri Lanka. Conservation status of the fauna and flora. Colombo, Sri Lanka; 2012 | 1 | 0 |
| Missouri Botanical Garden. Flora of the Jatun Sacha Biological Station. Preliminary Checklist; 2015. Available: http://www.mobot.org/MOBOT/research/ecuador/jatun/checklist.shtml. Accessed 15 January 2015 | 1 | 0 |
| Nakamura K, Suwa R, Denda T, Yokota M. Geohistorical and current environmental influences on floristic differentiation in the Ryukyu Archipelago, Japan. Journal of Biogeography. 2009; 36: 919–928. doi: 10.1111/j.1365-2699.2008.02057.x | 1 | 0 |
| Nationalpark Eifel. Artenliste Farne und Blütenpflanzen; 2015. Available: http://www.nationalpark-eifel.de/go/artenliste.html. Accessed 11 March 2015 | 1 | 0 |
| Nee M. Flora de la Región del Parque Nacional Amboró, Bolivia; 2015. Available: http://www.nybg.org/botany/nee/ambo/List.html. Accessed 12 January 2015 | 1 | 0 |
| Nikolić T. Flora Croatica Database; 2016. Available: http://hirc.botanic.hr/fcd. Accessed 12 February 2016 | 1 | 0 |
| Norton J, Majid SA, Allan D, Al Safran M, Böer B, Richer RA. An illustrated checklist of the flora of Qatar. Gosport, UK: Browndown Publications Gosport; 2009 | 1 | 1 |
| Notov AA. National park “Zavidovo”: vascular plants, bryophyte, lichens. Moscow; 2010 | 1 | 0 |
| NPS. NPSpecies. Information on Species in National Parks; 2015. Available: https://irma.nps.gov/NPSpecies/. Accessed 9 April 2015 | 1 | 0 |
| Oggero AJ, Arana MD. Inventario de las plantas vasculares del sur de la zona serrana de Córdoba, Argentina. Hoehnea. 2012; 39: 171–199 | 1 | 0 |
| Oliveira-Filho AT. NeoTropTree. Flora arbórea da Região Neotropical: Um banco de dados envolvendo biogeografia, diversidade e conservação; 2014. Available: http://www.icb.ufmg.br/treeatlan/. Accessed 12 February 2016 | 0 | 1 |
| Owiunji I, Nkuutu D, Kujirakwinja D, Liengola I, Plumptre AJ, Nsanzurwimo A, et al. The biodiversity of the Virunga Volcanoes | 1 | 0 |
| Pal D, Kumar A, Dutt B. Floristic diversity of Theog Forest Division, Himachal Pradesh, Western Himalaya. Check list. 2014; 10: 1083–1103 | 1 | 1 |
| Pandža M, Milović M, Kripina, Tafra D. Vascular flora of the Vrgada islets (Zadar Archipelago, Eastern Adriatic). Nat Croat. 2011; 20: 97–116 | 0 | 1 |
| Pandža M, Milović M. Flora of the islets near Pakoštane (Dalmatia, Croatia). Nat Croat. 2015; 24: 19–35. doi: 10.20302/NC.2015.24.2 | 0 | 1 |
| Pandža M, Milović M. Flora of the Veliki Lagan and Mali Lagan islets (Dugi Otok island, Croatia). Nat Croat. 2015; 24: 215–222. doi: 10.20302/NC.2015.24.13 | 0 | 1 |
| Pandža M, Skvorc Z. The flora of some uninhabited Sibenik Archipelago islands (Dalmatia, Croatia). Nat Croat. 2002; 11: 367–385 | 1 | 1 |
| Pandža M. Flora of the island of Zirje and the small islands around it (eastern Adriatic coast, Croatia). Acta Botica Croatia. 2003; 62: 115–139 | 1 | 1 |
| Pandža M. Flora of the small islands of Murter. Nat Croat. 2002; 11: 77–101 | 1 | 1 |
| Pandža M. Flora parka prirode Papuk (Slavonija, Hrvatska). Šumarski list. 2010; 134: 25–43 | 1 | 1 |
| Parks Canada. Biotics Web Explorer; 2015. Available: http://www.pc.gc.ca/apps/bos/BOSIntro_e.asp. Accessed 13 April 2015 | 1 | 0 |
| Paula S, Arianoutsou M, Kazanis D, Tavsanoglu Ç, Lloret F, Buhk C, et al. Fire‐related traits for plant species of the Mediterranean Basin. Ecology. 2009; 90: 1420 | 0 | 1 |
| Pawlaczyk J, Pawlaczyk P. Vascular plants of Drawa National park and its neighbourhood; 2000. Available: http://www.eko.org.pl/lkp/dpn/chckl_rosliny_sh.html. Accessed 23 April 2015 | 1 | 0 |
| Pedashenko H, Vassilev K. Flora of Ponor Special Protection Area (Natura 2000), western Bulgaria. Acta zoologica bulgarica. 2014; 5: 33–60 | 1 | 1 |
| Peña-Chocarro MdC. Updated checklist of vascular plants of the Mbaracayú Forest Nature Reserve (Reserva Natural del Bosque Mbaracayú), Paraguay. Auckland, N.Z.: Magnolia Press; 2010 | 1 | 1 |
| Pôle Flore Habitats. Catalogue de la flore vasculaire de Rhône-Alpes; 2015. Available: http://www.pifh.fr/pifhcms/index.php. Accessed 12 January 2015 | 1 | 0 |
| Programma de Conservación y Manejo. Parque Nacional Cumbres de Monterrey. Mexico; 2006 | 1 | 0 |
| Queensland Government. Census of the Queensland flora 2014; 2014. Available: https://data.qld.gov.au/dataset/census-of-the-queensland-flora-2014. Accessed 5 February 2015 | 1 | 0 |
| Rahman AHMM. Angiospermic Flora of Rajshahi District, Bangladesh. AJLS. 2013; 1: 105 | 0 | 1 |
| Rahman MS, Hossain GM, Khan SA, Uddin SN. An annotated Checklist of the Vascular Plants of Sundarban Mangrove Forest of Bangladesh. Bangladesh Journal of Plant Taxonomy. 2015; 22: 17–41 | 1 | 1 |
| Rakov NS, Saksonov S.V., Senator S.А., Vasjukov V.M. Vascular plants of Ulyanovsk Region. Togliatti: Russian Academy of Sciences; 2014 | 1 | 0 |
| Robinson AC, Canty PD, Fotheringham D. Investigator group expedition 2006. flora and vegetation. Trans. R. Soc. S. Aust. 2008; 132: 173–220 | 1 | 0 |
| Robinson AC, Canty PD, Wace NM, Barker RM. The encounter 2002 expedition to the isles of St Francis, South Australia. flora and vegetation. Trans. R. Soc. S. Aust. 2003; 127: 107–128 | 1 | 0 |
| Rossetto EFS, Vieira AOS. Vascular Flora of the Mata dos Godoy State Park, Londrina, Paraná, Brazil. Check list. 2013; 9: 1020–1034 | 1 | 1 |
| Royal Botanic Gardens and Domain Trust. PlantNET - The NSW Plant Information Network System; 2017. Available: http://plantnet.rbgsyd.nsw.gov.au. Accessed 16 September 2016 | 1 | 1 |
| Rundel PW, Dillon MO, Palma B. Flora and Vegetation of Pan de Azúcur National Park in the Atacama desert of Northern Chile. Gayana Bot. 1996; 53: 295–315 | 1 | 1 |
| SANBI. Plants of Southern Africa. An online checklist; 2014. Available: http://posa.sanbi.org. Accessed 9 March 2015 | 1 | 1 |
| Sandbakk BE, Alsos IG, Arnesen G, Elven R. The flora of Svalbard; 1996. Available: http://svalbardflora.no/. Accessed 16 March 2011 | 1 | 0 |
| Schaefer H, Hardy OJ, Silva L, Barraclough TG, Savolainen V. Testing Darwin's naturalization hypothesis in the Azores. Ecol Lett. 2011; 14: 389–396. doi: 10.1111/j.1461-0248.2011.01600.x | 0 | 1 |
| Schönfelder P, Schönfelder I. Die Kosmos-Kanarenflora. Über 850 Arten der Kanarenflora und 48 tropische Ziergehölze. Stuttgart: Franckh-Kosmos; 1997 | 0 | 1 |
| Scouppe M. Composition floristique et diversité de la végétation de la zone Est du Parc National de Taï (Côte d’Ivoire). Master, Université de Genève. 2011 | 1 | 1 |
| Searle J, Madden S. Flora assessment of South Stradbroke Island. Gold Coast City, Australia: Gold Coast City Council; 2006 | 1 | 0 |
| SEINet. SEINet Flora Projects; 2015. Available: http://swbiodiversity.org/seinet/projects/index.php. Accessed 2 April 2015 | 1 | 0 |
| Selvi F. A critical checklist of the vascular flora of Tuscan Maremma (Grosseto province, Italy). Fl. Medit. 2010; 20: 47–139 | 1 | 1 |
| Senterre B, Chew MY, Chung RCK. Flora and vegetation of Pulau Babi Tengah, Johor, Peninsular Malaysia. Check list. 2015; 11: 1714. doi: 10.15560/11.4.1714 | 0 | 1 |
| Seregin AP. New Flora of the Maeshchera National Park (Vladimir Oblast, Russia). Checklist, distribution atlas, peculiarities, and distributional changes in species over the last decade (2002–2012). Tula: ASTRA; 2013 | 1 | 0 |
| Shaheen H, Qureshi R, Akram A, Gulfraz M, Potter D. A preliminary floristic checklist of Thal Desert Punjab, Pakistan. Pakistan Journal of Botany. 2014; 46: 13–18 | 1 | 0 |
| Shaw JD, Spear D, Greve M, Chown SL. Taxonomic homogenization and differentiation across Southern Ocean Islands differ among insects and vascular plants. J Biogeogr. 2010; 37: 217–228 | 1 | 0 |
| Shherbina S. Flora of Vascular Plants of Central Siberian State Biosperic Reserve and neighboring territories. Turczaninowia. 2009; 12: 71–241 | 1 | 0 |
| Short PS, Albrecht DE, Cowie ID, Lewis DL, Stuckey BM. Checklist of the vascular plants of the Northern Territory. Darwin: Department of Natural Resources, Environment, The Arts and Sport; 2011 | 1 | 0 |
| Silaeva TB, Chugunov GG, Kiryukhin IV, Ageeva AM, Vargot EV, Grishutkina GA, et al. Flora of the national park "Smolny". Mosses and vascular plants: annotated list of species [In Russian]: Commission of RAS for the Conservation of Biological Diversity [Комиссия РАН по сохранению биологического разнообразия] | 1 | 0 |
| Singh A. Observations on the vascular flora of Banaras Hindu University Main Campus, India. International Journal of Modern Biology and Medicine. 2015; 6: 48–87 | 1 | 1 |
| Skelin M, Ljubičić I, Skelin I, Vitasović Kosić I, Bogdanović S. The flora of Zečevo (Hvar Archipelago, Croatia). Agriculturae Conspectus Scientificus. 2014; 79: 85–91 | 0 | 1 |
| Slik FJW, Arroyo-Rodríguez V, Aiba S-I, Alvarez-Loayza P, Alves LF, Ashton P, et al. An estimate of the number of tropical tree species. Proceedings of the National Academy of Sciences of the United States of America. 2015; 112: 7472–7477. doi: 10.1073/pnas.1423147112 | 0 | 1 |
| SLUFG. Rote Liste und Artenliste Sachsen. Farn- und Samenpflanzen. Dresden: Sächsisches Landesamt für Umwelt, Landwirtschaft und Geologie; 2015 | 1 | 0 |
| Smith AC. Flora Vitiensis nova. a new Flora of Fiji (spermatophytes only): Pacific Tropical Botanical Garden (Lawaii, Hawaii); 1979-1996 | 0 | 1 |
| Smithsonian Institution. A checklist of Trees, Shrubs, Herbs, and Climbers of Myanmar. Contributions from the United States National Herbarium. 2003; 45: 1–590 | 1 | 0 |
| Stace CA, Ellis RG, Kent DH, McCosh DJ. Vice-county Census Catalogue of the vascular plants of Great Britain, the Isle of Man and the Channel Islands. London, UK: Botanical Society of the British Isles; 2003 | 1 | 0 |
| Stace CA, Ellis RG, Kent DH, McCosh DJ. Vice-county Census Catalogue of the vascular plants of Great Britain, the Isle of Man and the Channel Islands. London, UK: Botanical Society of the British Isles; 2003 | 1 | 0 |
| Stalmans M. Tinley's plant species list for the Greater Gorongosa ecosystem, Moçambique. Unpublished report by International Conservation Services to the Carr Foundation and the Ministry of Tourism; 2006 | 1 | 1 |
| Stalter R, Lamont EE. The historical and extant flora of Sable Island, Nova Scotia, Canada. Journal of the Torrey Botanical Society. 2006; 133: 362–374 | 1 | 0 |
| Tamis WLM, van der Meijden R, Runhaar J, Bekker RM, Ozinga WA, Odé B, et al. Standard List of the Flora of the Netherlands 2003. Gorteria. 2004; 30: 101–195 | 1 | 1 |
| Tatewaki M. Geobotanical studies on the Kurile Islands. Acta Horti Gotoburgensis. 1957; 21: 43–123 | 1 | 0 |
| Ter Steege H, Vaessen RW, Cardenas-Lopez D, Sabatier D, Antonelli A, Oliveira SM de, et al. The discovery of the Amazonian tree flora with an updated checklist of all known tree taxa. Sci Rep. 2016; 6: 29549. doi: 10.1038/srep29549 | 0 | 1 |
| Thiombiano A, Schmidt M, Dressler S, Ouédraogo A, Hahn K. Catalogue des plantes vasculaires du Burkina Faso. Boissiera: mémoires des Conservatoire et Jardin botaniques de la Ville de Genève. 2012; 65: 1–391 | 1 | 1 |
| Thomas J. Plant diversity of Saudi Arabia. Flora checklist; 2011. Available: http://plantdiversityofsaudiarabia.info/biodiversity-saudi-arabia/flora/Checklist/Cheklist.htm. Accessed 14 January 2016 | 1 | 0 |
| Tonkov S, Pavlova D, Atanassova J, Nedelcheva A, Marinova E. Floristis catalogue of the nature reserve Rilomanastirska Gora (Central Rila Mountains). I. The locality Kirilova: University of Sofia; 2004 | 0 | 1 |
| Tressens SG, Keller HA, Revilla V. Las plantas vasculares de la reserva de uso múltiple Guaraní, Misiones (Argentina). Boletín de la Sociedad Argentina de Botánica. 2008; 43: 273–293 | 1 | 0 |
| Tropicos. Catálogo de las Plantas Vasculares de Bolivia. Tropicos. St. Louis: Missouri Botanical Garden; 2015 | 1 | 1 |
| Tropicos. Catalogue of the Vascular Plants of Ecuador; 2015. Available: http://www.tropicos.org/Project/CE. Accessed 22 October 2015 | 1 | 1 |
| Tropicos. Catalogue of the Vascular Plants of the Department of Antioquia (Colombia). Tropicos. St. Louis: Missouri Botanical Garden; 2015 | 1 | 1 |
| Tropicos. Flora de Nicaragua. Tropicos. St. Louis: Missouri Botanical Garden; 2015 | 1 | 0 |
| Tropicos. Listado de la Flora del Parque Nacional Madidi, Bolivia. Tropicos. St. Louis: Missouri Botanical Garden; 2015 | 1 | 0 |
| Tropicos. Panama Checklist. Tropicos. St. Louis: Missouri Botanical Garden; 2015 | 1 | 1 |
| Tropicos. Paraguay Checklist. Tropicos. St. Louis: Missouri Botanical Garden; 2015 | 0 | 1 |
| Tropicos. Peru Checklist. Tropicos. St. Louis: Missouri Botanical Garden; 2015 | 1 | 1 |
| Tropicos. Peru Checklist. Tropicos. St. Louis: Missouri Botanical Garden; 2015 | 1 | 1 |
| Turner IM. A catalogue of the vascular plants of Malaya. Singapore: Gardens' Bulletin; 1995 | 1 | 1 |
| Tutul E, Uddin MZ, Rahman MO, Hassan MA. Angiospermic flora of Runctia sal forest, Bangladesh. I. Liliopsida (Monocots). Bangladesh Journal of Plant Taxonomy. 2009; 16: 83–90 | 0 | 1 |
| UIB. Herbario virtual del Mediterráneo Occidental; 2007. Available: http://herbarivirtual.uib.es/cas-med/. Accessed 7 August 2012 | 0 | 1 |
| University of Greifswald. FloraGREIF - Virtual Flora of Mongolia; 2010. Available: http://greif.uni-greifswald.de/floragreif/. Accessed 2 February 2016 | 0 | 1 |
| USDA, NRCS. The PLANTS Database; 2015. Available: http://plants.usda.gov. Accessed 24 April 2015 | 1 | 1 |
| van Vreeswyk AME, Payne AL, Leighton KA, Hennig P. An inventory and condition survey of the Pilbara region, Western Australia: Department of Agriculture; 2004 | 1 | 1 |
| Vanderplank SE. The Vascular Flora of Greater San Quintín, Baja California, Mexico. CGU Theses & Dissertations; 2010 | 1 | 1 |
| Veklich TN. Flora of the Norsky Nature Reserve (Amur region). Blagoveshensk: Russian Academy of Sciences, Far East Division; 2009 | 1 | 0 |
| Velarde E, Wilder BT, Felcer RS, Ezcurra E. Floristic diversity and dynamics of Isla Rasa, Gulf of California - A globally important seabird island. Botanical Sciences. 2014; 92: 89–101 | 0 | 1 |
| Velarde EP, Guzmán RC, Koch SD. Plantas vasculares y vegetación de la parte alta del Arroyo Agua Fría, municipio de Minatitlán, Colima, México. Acta Botanica Mexicana. 2008; 84: 25–72 | 1 | 1 |
| Velayos Rodríguez M. Flora de Guinea Equatorial; 2016. Available: http://www.floradeguinea.com/. Accessed 14 February 2016 | 1 | 0 |
| VicFlora. Flora of Victoria; 2016. Available: http://vicflora.rbg.vic.gov.au. Accessed 30 November 2016 | 1 | 1 |
| Viciani D, Gonnelli V, Sirotti M, Agostini N. An annotated check-list of the vascular flora of the “Parco Nazionale delle Foreste Casentinesi, Monte Falterona e Campigna”(Northern Apennines Central Italy). Webbia. 2010; 65: 3–131 | 1 | 1 |
| Vogt C. Composición de la Flora Vascular del Chaco Boreal, Paraguay. I. Pteridophyta y Monocotiledoneae. Steviana. 2011; 3: 13–47 | 1 | 1 |
| Wagner WL, Herbst DR, Lorence DH. Flora of the Hawaiian Islands website; 2005. Available: http://botany.si.edu/pacificislandbiodiversity/hawaiianflora/. Accessed 16 October 2010 | 0 | 1 |
| WCSP. World Checklist of Selected Plant Families; 2014. Available: http://apps.kew.org/wcsp/home.do. Accessed 1 December 2014 | 0 | 1 |
| Webster GL, Rhode RM. Plant diversity of an Andean cloud forest: inventory of the vascular plants of Maquipucuna, Ecuador. Publications in Botany. 2001; 82: 1–228 | 1 | 1 |
| Wellington Botanical Society. Native vascular plants of Great Barrier Island. Wellington, New Zealand: Wellington Botanical Society; 2008 | 1 | 0 |
| Western Australian Herbarium. FloraBase - the Western Australian Flora; 2017. Available: https://florabase.dpaw.wa.gov.au/. Accessed 16 September 2016 | 1 | 1 |
| Wieringa JJ. Flora of Gabon. unpublished; 23.04.2016 | 0 | 1 |
| Woodroffe CD. Vascular plant speciesarea relationships on Nui Atoll, Tuvalu, Central Pacific: a reassessment of the small island effect. Australien Journal of Ecology. 1986; 11: 21–31 | 0 | 1 |
| Yakubov VV. Illustrated Flora of the Kronotsky Reserve (Kamchatka): Vascular plants. Vladivostok: Institute of Biology and Soil Science; 2010 | 1 | 0 |
| Yineger H, Kelbessa E, Bekele T, Lulekal E. Floristic composition and structure of the dry afromontane forest at Bale Mountains National Park, Ethiopia. SINET: Ethiopian Journal of Science. 2008; 31: 103–120 | 1 | 1 |
| ZDSF, SKEW. Info Flora. Artenliste Schweiz 5x5 km; 2014. Available: https://www.infoflora.ch/de/daten-beziehen/artenliste-5x5-km.html. Accessed 13 February 2015 | 1 | 0 |
| Zhang ST, Zhen Du G, Chen JK. Seed size in relation to phylogeny, growth form and longevity in a subalpine meadow on the east of the Tibetan plateau. Folia Geobot. 2004; 39: 129–142. doi: 10.1007/BF02805242 | 0 | 1 |
| Zuloaga FO, Morrone O, Belgrano M. Catálogo de las Plantas Vasculares del Cono Sur; 2014. Available: http://www.darwin.edu.ar/Proyectos/FloraArgentina/fa.htm. Accessed 16 March 2015 | 1 | 1 |
| Zvyagintseva KO. An annotated checklist of the urban flora of Kharkiv. Kharkiv, Ukraine: Kharkiv National University; 2015 | 1 | 1 |
| Абрамова ЛА, Волкова ПА. Сосудистые растения Байкальского заповедника. (Аннотированный список видов). Флора и фауна заповедников. 2011; 117: 1–112 | 1 | 0 |
| Антипова EM. Флора внутриконтинентальных островных лесостепей Средней Сибири. Красноярск: гос. пед. ун-т им. В.П. Астафьева; 2012 | 1 | 0 |
| Артемов ИА. Определитель растений Катунского биосферного заповедника. БАРНАУЛ: Russian Academy of Sciences; 2012 | 1 | 1 |
| Гаджиев ВД, Юсифов ЭФ. Флора и растительность Кызылагачского заповедника и их биоразнообразие. Баку: Национальная Академия Наук Азербайджана; 2003 | 1 | 0 |
| Евстигнеев ОИ, Федотов ЮП. Флора сосудистых растений заповедника "Брянский лес". Брянск: Гос. природ. биосфер. заповедник Брян. лес; 2007 | 1 | 0 |
| Куликов ПВ, Кирсанова ОФ. Сосудистые растения заповедника "Денежкин камень". (Аннотированный список видов). Флора и фауна заповедников. 2012; 119: 1–140 | 1 | 0 |
| Лактионов АП, Пилипенко ВН, Глаголев СБ, Лактионова НА. Сосудистые растения заповедника «Богдинско-Баскунчакский» (Аннотированный список видов): Флора и фауна заповедников. Флора и фауна заповедников. 2008; 113: 1–113 | 1 | 0 |
| Миркина БМ. Флора и растительность Национального парка «Башкирия»: Russian Academy of Sciences; 2010 | 1 | 0 |
| Морозова ОВ, Царевская НГ, Белоновская ЕА. Сосудистые растения национального парка «Валдайский». Флора и фауна заповедников. 2010; 7: 1–98 | 1 | 0 |
| Хапугин AA. Сосудистые растения Ромодановского района Республики Мордовия (конспект флоры). Saransk; 2013 | 1 | 0 |
