# Supplementary material for: Biodiversity data integration—the significance of data resolution and domain
Source: PLoS Biol. 2019 Mar 18;17(3):e3000183. doi: 10.1371/journal.pbio.3000183 (PMC6445469; doi:10.1371/journal.pbio.3000183)
Supplement: S2 Data — (ZIP) [file pbio.3000183.s005.zip › S2_references.docx]

| **Reference** | **checklist** | **traits** |
| --- | --- | --- |
| 3D Environmental. Profile for management of the habitats and related ecological and cultural resource values of Boigu Island; 2013 | 0 | 1 |
| Abdel Khalik K, El-Sheikh M, El-Aidarous A. Floristic diversity and vegetation analysis of Wadi Al-Noman, Mecca, Saudi Arabia. Turkish Journal of Botany. 2013; 37: 894–907 | 0 | 1 |
| Abderrahman A, El Kadmiri AA. Richesse et diversité floristique de la subéraie de la Mamora (Maroc). Acta Botanica Malacitana. 2005; 30: 127–138 | 1 | 0 |
| Adou Yao CY. Diversité floristique et végétation dans le Parc National de Taï, Côte d'Ivoire. Abidjan: Tropenbos Côte d'Ivoire; 2005 | 1 | 0 |
| Alves, Ruy José Válka, Kolbek J. Summit vascular flora of Serra de São José, Minas Gerais, Brazil. Check list. 2009; 5: 35–73 | 0 | 1 |
| Arakaki M, Cano A. Composición florística de la cuenca del río Ilo-Moquegua y Lomas de Ilo, Moquegua, Peru. Revista Peruana de Biología. 2003; 10: 5–19 | 0 | 1 |
| Assédé EPS, Adomou AC, Sinsin B. Magnoliophyta, Biosphere Reserve of Pendjari, Atacora Province, Benin. Check list. 2012; 8: 642–661 | 0 | 1 |
| Azbukina ZM, Bardunov LV, Bezdeleva TA, Bogacheva AV, Bulakh EM, Vasilyeva LN, et al. Flora, vegetation and mycobiota of the reserve «Ussuriysky». Vladivostok: Dalnauka; 2006 | 1 | 0 |
| Badshah L, Hussain F, Sher Z. Floristic inventory, ecological characteristics and biological spectrum of rangeland, District Tank, Pakistan. Pak. J. Bot. 2013; 45: 1159–1168 | 1 | 1 |
| Bakis Y, Babac MT, Uslu E. Tübives. Turkish Plants Data Service; 2015. Available: http://www.tubives.com/. Accessed 13 February 2015 | 1 | 0 |
| Bancheva S, Vassilev K. Vascular flora of the Beli Lom Nature Reserve in Northeast Bulgaria. Phytologia Balcanica. 2006; 12: 377–386 | 1 | 1 |
| Barker WR, Barker RM, Jessop JP, Vonow HP. Census of South Australian vascular plants. Journal of the Adelaide Botanic Gardens Supplement. 2005; 1: 1–396 | 1 | 0 |
| Benito BM, Lorite J, Pérez-Pérez R, Gómez-Aparicio L, Peñas J, Robertson M. Forecasting plant range collapse in a mediterranean hotspot. When dispersal uncertainties matter. Diversity Distrib. 2014; 20: 72–83. doi: 10.1111/ddi.12148 | 0 | 1 |
| Bernal R, Gradstein SR, Celis M. Catálogo de plantas y líquenes de Colombia; 2015. Available: http://catalogoplantasdecolombia.unal.edu.co/. Accessed 15 January 2016 | 1 | 1 |
| BGCI. GlobalTreeSearch online database; 2017. Available: www.bgci.org/globaltree_search.php. Accessed 14 August 2017 | 0 | 1 |
| Bingham MG, Willemen A, Wursten BT, Ballings P, Hyde MA. Flora of Zambia; 2016. Available: http://www.zambiaflora.com/. Accessed 14 November 2016 | 1 | 0 |
| BioScripts. Flora Vascular; 2014. Available: http://www.floravascular.com/. Accessed 25 May 2014 | 1 | 1 |
| Botanical Garden Tel Aviv. Israel Flora: Tel Aviv University | 1 | 1 |
| Breckle S-W, Hedge IC, Rafiqpoor MD. Vascular plants of Afghanistan. An augmented checklist. Bonn: Scientia Bonnensis; 2013 | 1 | 0 |
| Brennan K. An annotated checklist of the vascular plants of the Alligator Rivers Region, Northern Territory, Australia. Barton, Australia: Supervising Scientist; 1996 | 0 | 1 |
| Bridgewater SGM, Harris DJ, Whitefoord C, Monro AK, Penn MG, Sutton DA, et al. A Preliminary Checklist of the vascular plants of the Chiquibul Forest, Belize. Edin. Jnl of Bot. 2006; 63: 269–321 | 1 | 1 |
| Brundu G, Camarda I. The Flora of Chad: a checklist and brief analysis. Phytokeys. 2013; 23: 1–17 | 1 | 1 |
| Bundesamt für Naturschutz. Floraweb; 2016. Available: www.floraweb.de. Accessed 15 September 2016 | 1 | 1 |
| Cascante-Marín A, Estrada-Chavarría A. Las plantas vasculares de El Rodeo, Costa Rica. Brenesia. 2012; 77: 71–128 | 0 | 1 |
| Catarino L, Martins ES, Basto MF, Diniz MA. An annotated checklist of the vascular flora of Guinea-Bissau (West Africa). Blumea-Biodiversity, Evolution and Biogeography of Plants. 2008; 53: 1–222 | 1 | 1 |
| Chang C-S, Kim H, Chang K. Provisional Checklist of the Vascular Plants for the Korea Peninsular Flora (KPF). Version 1.0. Korea | 1 | 0 |
| Chawla A, Parkash O, Sharma V, Rajkumar S, Lal B, Gopichand, et al. Vascular plants, Kinnaur, Himachal Pradesh, India. Check list. 2012; 8: 321–348 | 1 | 1 |
| Chinese Virtual Herbarium. The Flora of China v. 5.0; 2016. Available: http://www.cvh.org.cn/. Accessed 15 January 2016 | 1 | 1 |
| Chong KY, Tan TWH, Corlett RT. A checklist of the total vascular plant flora of Singapore. Native, Naturalised and Cultivated Species. Singapore: Raffles Museum of Biodiversity Research; 2009 | 0 | 1 |
| Clark JL, Neill DA, Asanza M. Floristic checklist of the Mache-Chindul mountains of Northwestern Ecuador. Contributions from the United States National Herbarium. 2006; 54: 1–180 | 0 | 1 |
| Cochard R, Bloesch U. Electronic plant species database of the Saadani National Park, coastal Tanzania; 2007. Available: http://www.wildlife-baldus.com/saadani.html. Accessed 14 November 2016 | 1 | 1 |
| CONABIO. Sistema Nacional de Información sobre Biodiversidad; 2016. Available: https://www.gob.mx/conabio. Accessed 11 April 2016 | 1 | 0 |
| Conti F, Abbate G, Alessandrini A, Blasi C. Annotated Checklist of the Italian Vascular Flora. Italy: Palombi Editori, Roma; 2005 | 1 | 0 |
| Costion C, Lorence D. The endemic plants of Micronesia. A geographical checklist and commentary. Micronesica. 2012; 43: 51–100 | 0 | 1 |
| Cuc Phuong National Park. Flora of the Cuc Phuong National Park. Vietnam; 2011 | 1 | 0 |
| Da Vela M, Frignani F, Bonari G, Angiolini C. La flora vascolare della diserva naturale "La Pietra" (Toscana meridionale). Micol. Veget. Medit. 2013; 28: 135–160 | 0 | 1 |
| Danihelka J, Chrtek J, Kaplan Z. Checklist of vascular plants of the Czech Republic. Preslia. 2012; 84: 647–811 | 1 | 0 |
| Dauby G, Leal M, Stevart T. Vascular plant checklist of the coastal National Park of Pongara, Gabon. Systematics and geography of plants. 2008; 78: 155–216 | 1 | 1 |
| Dauby G, Zaiss R, Blach-Overgaard A, Catarino L, Damen T, Deblauwe V, et al. RAINBIO. A mega-database of tropical African vascular plants distributions. PK. 2016; 74: 1–18. doi: 10.3897/phytokeys.74.9723 | 1 | 1 |
| de la Luz, León, Rebman J, Domínguez-León M, Domínguez-Cadena R. The vascular flora and floristic relationships of the sierra de la Giganta in Baja California Sur, México. Revista mexicana de biodiversidad. 2008; 79: 29–65 | 0 | 1 |
| Desmet P, Brouillet L. Database of Vascular Plants of Canada (VASCAN): a community contributed taxonomic checklist of all vascular plants of Canada, Saint Pierre and Miquelon, and Greenland. Phytokeys. 2013; 25: 55–67 | 0 | 1 |
| Dimopoulos P, Raus T, Bergmeier E, Constantinidis T, Iatrou G, Kokkini S, et al. Vascular plants of Greece. An annotated checklist. Berlin, Athens: Botanischer Garten und Botanisches Museum Berlin-Dahlem, Freie Universität Berlin; Hellenic Botanical Society; 2013 | 1 | 1 |
| Domínguez E, Marticorena C, Elvebakk A, Pauchard A. Catálogo de la flora vascular del Parque Nacional Pali Aike. XII Región, Chile. Gayana Botánica. 2004; 61: 67–72 | 0 | 1 |
| Doroftei M, Oprea A, Ştefan N, Sârbu I. Vascular wild flora of Danube Delta Biosphere Reserve. Sci. Annals of Danube Delta Institute. 2011; 17: 15–52 | 1 | 0 |
| Egea J de, Peña-Chocarro M, Espada C, Knapp S. Checklist of vascular plants of the Department of Ñeembucú, Paraguay. Phytokeys. 2012; 9: 15–179 | 1 | 1 |
| Ejaz-ul-Islam Dar M, Cochard R, Shrestha RP, Ahmad S. Floristic composition of Machiara National Park, District Muzaffarabad Azad Kashmir, Pakistan. International Journal of Biosciences. 2012; 2: 28–45 | 1 | 1 |
| El-Ghani MM, Abdel-Khalik KN. Floristic Diversity and Phytogeography of the Gebel Elba National Park, South-East Egypt. Turkish Journal of Botany. 2005; 30: 121–136 | 1 | 1 |
| Engemann K, Sandel B, Boyle BL, Enquist BJ, Jørgensen PM, Kattge J, et al. A plant growth form dataset for the New World. Ecology. 2016; 97: 3243. doi: 10.1002/ecy.1569 | 0 | 1 |
| Espinosa-Jiménez JA, Pérez-Farrera MÁ, Martínez-Camilo R. Inventario florístico del Parque Nacional Cañón del Sumidero, Chiapas, México. Boletín de la Sociedad Botánica de México. 2011; 89: 37–82 | 0 | 1 |
| Evarts-Bunders P, Evarte-Bundere G, Bāra J, Nitcis M. The flora of vascular plants in nature reserve „Eglone”. Acta Biologica Universitatis Daugavpiliensis. 2013; 13: 21–38 | 1 | 0 |
| Fern K, Fern A. Useful Tropical Plants Database; 2015. Accessed 25 July 2015 | 0 | 1 |
| Ferreira AL, Coutinho BR, Pinheiro HT, Thomaz LD. Composição florística e formações vegetais da Ilha dos Franceses, Espírito Santo. Bol. Mus. Biol. Mello Leitao. 2007; 22: 25–44 | 0 | 1 |
| Figueiredo E, Paiva J, Stevart T, Oliveira F, Smith GF. Annotated catalogue of the flowering plants of São Tomé and Príncipe. Bothalia. 2011; 41: 41–82 | 0 | 1 |
| Figueroa-C. Y, Galeano G. Lista comentada de las plantas vasculares del enclave seco interandino de La Tatacoa (Huila, Colombia). Caldasia. 2007; 29: 263–281 | 0 | 1 |
| Fischer E, Rembold K, Althof A, Obholzer J, Malombe I, Mwachala G, et al. Annotated checklist of the vascular plants of Kakamega Forest, Western Province, Kenya. Journal of East African Natural History. 2010; 99: 129–226 | 1 | 1 |
| Fischer MA, Adler W, Oswald K. Exkursionsflora für Österreich, Liechtenstein und Südtirol. Bestimmungsbuch für alle in der Republik Österreich, im Fürstentum Liechtenstein und in der Autonomen Provinz Bozen. 3rd ed. Linz: OÖ Landesmuseum; 2008 | 1 | 1 |
| Funk VA, Hollowell T, Berry P, Kelloff C, Alexander SN. Checklist of the plants of the Guiana Shield (Venezuela: Amazonas, Bolivar, Delta Amacuro; Guyana, Surinam, French Guiana). Washington, DC: Department of Botany, National Museum of Natural History; 2007 | 1 | 0 |
| Gafurova ММ. Vascular plants of Chuvash Republic. Flora of the Volga River Basin V. III: The Russian Academy of Sciences; 2014 | 1 | 0 |
| Gamit SB, Maurya RR, Qureshimatva UM, Solanki HA. Check list of flowering plants in Tapi District, Gujarat, India. International Journal of advanced Research. 2015; 3: 1104–1123 | 0 | 1 |
| Gnoumou A, Ouedraogo O, Schmidt M, Thiombiano A. Floristic diversity of classified forest and partial faunal reserve of Comoé-Léraba, southwest Burkina Faso. Check list. 2015; 11: 1557 | 0 | 1 |
| Grozeva NH. The flora of Atanasovsko lake natural reserve. In: Gruev B, Nikolova M, Donev A, editors. Proceedings of the Balkan Scientific Conference of Biology; 2005. pp. 381–396 | 1 | 0 |
| Harris DJ. The vascular plants of the Dzanga-Sangha Reserve, Central African Republic. Edinburgh, Scotland, UK: Royal Botanic Garden Edinburgh; 2002 | 0 | 1 |
| Hauenstein E, Muñoz-Pedreros A, Yánez J, Sánchez P, Möller P, Guiñez B, et al. Flora y vegetación de la Reserva Nacional Lago Peñuelas, Reserva de la Biósfera, Región de Valparaíso, Chile. Bosque (Valdivia). 2009; 30: 159–179. doi: 10.4067/S0717-92002009000300006 | 0 | 1 |
| Hawkins BA, Rueda M, Rangel TF, Field R, Diniz-Filho JAF, Linder P. Community phylogenetics at the biogeographical scale: cold tolerance, niche conservatism and the structure of North American forests. J. Biogeogr. 2013; 41: 23–38 | 0 | 1 |
| Heberling JM, Jo I, Kozhevnikov A, Lee H, Fridley JD. Biotic interchange in the Anthropocene. Strong asymmetry in East Asian and eastern North American plant invasions. Global Ecol. Biogeogr. 2017; 26: 447–458. doi: 10.1111/geb.12551 | 0 | 1 |
| Hoang VS. Uses and conservation of plant diversity in Ben En National Park, Vietnam. Ph.D., National Herbarium of the Netherlands, Leiden University Branch. 2009 | 1 | 1 |
| Hyde MA, Wursten BT, Ballings P, Coates Palgrave M. Flora of Botswana; 2016. Available: http://www.botswanaflora.com/. Accessed 14 November 2016 | 1 | 0 |
| Hyde MA, Wursten BT, Ballings P, Coates Palgrave M. Flora of Malawi; 2016. Available: http://www.malawiflora.com/index.php. Accessed 14 November 2016 | 1 | 0 |
| Hyde MA, Wursten BT, Ballings P, Coates Palgrave M. Flora of Mozambique; 2016. Available: http://www.mozambiqueflora.com/. Accessed 14 November 2016 | 1 | 0 |
| Hyde MA, Wursten BT, Ballings P, Coates Palgrave M. Flora of Zimbabwe; 2016. Accessed 14 November 2016 | 1 | 0 |
| ICIMOD. Hindu Kush-Himalayan (HKH) Conservation Portal; 2015. Available: http://www.icimod.org/hkhconservationportal/Initiatives.aspx. Accessed 21 April 2015 | 1 | 0 |
| Jardim Botânico do Rio de Janeiro. Flora do Brasil 2020 em construção; 2016. Available: http://floradobrasil.jbrj.gov.br/. Accessed 9 May 2016 | 1 | 1 |
| Jasprica N, Dolina K, Milović M. Plant taxa and communities on three islets in south Croatia, NE Mediterranean. Nat Croat. 2015; 24: 191–213. doi: 10.20302/NC.2015.24.12 | 0 | 1 |
| Jiménez JE, Juárez P, Díaz A. Checklist of the vascular flora of Reserva Biológica San Luis, Costa Rica. Check list. 2016; 12: 1859. doi: 10.15560/12.2.1859 | 0 | 1 |
| Jordano P. FRUBASE; 2008. Available: http://ebd10.ebd.csic.es/mywork/frubase/frubase.html. Accessed 26.06.15 | 0 | 1 |
| Kabuye CHS, Mungai GM, Mutangah JG. Flora of Kora National Reserve. In: Coe M, Collins NM, editors. Kora: An Ecological Inventory of the Kora National Reserve, Kenya. Kora Research Project 1982-85 : a Joint Venture Between the National Museums of Kenya and the Royal Geographical Society: Royal Geographical Society; 1986. pp. 57–104 | 1 | 1 |
| Kattge J, Diaz S, Lavorel S, Prentice IC, Leadley P, Bönisch G, et al. TRY – a global database of plant traits. Global Change Biology. 2011; 17: 2905–2935 | 0 | 1 |
| Kattge J, Diaz S, Lavorel S, Prentice IC, Leadley P, Bönisch G, et al. TRY – a global database of plant traits. Global Change Biology. 2011; 17: 2905–2935 | 0 | 1 |
| Kemenes A. Distribuição espacial da flora terrestre fanerogâmica do Parque Nacional Marinho de Abrolhos, BA. Revista Brasil. Bot. 2003; 26: 141–150 | 0 | 1 |
| Keppel G. Summary report on forests of the Mataqali Nadicake Kilaka, Kubulau district, Bua, Vanua Levu; 2005 | 0 | 1 |
| Kim C-S, Koh J-G, Moon M-O, Song G-P, Hyun H-J, Song K-M, et al. Flora and life form spectrum of Hallasan natural reserve, Korea. Journal of the Environmental Sciences. 2007; 16: 1257–1269 | 0 | 1 |
| Kleyer M, Bekker RM, Knevel IC, Bakker JP, Thompson K, Sonnenschein M, et al. The LEDA Traitbase: a database of life-history traits of the Northwest European flora. J Ecol. 2008; 96: 1266–1274 | 0 | 1 |
| Klimeš L, Dickoré B. Flora of Ladak (NW Himalaya). A preliminary check-list; 2015. Available: http://www.butbn.cas.cz/klimes/desert.html. Accessed 12 January 2015 | 1 | 0 |
| Kraaij T. The flora of the Bontebok National Park in regional perspective. South African Journal of Botany. 2011; 77: 455–473. doi: 10.1016/j.sajb.2010.09.013 | 0 | 1 |
| Kraft TS, Wright SJ, Turner I, Lucas PW, Oufiero CE, Supardi Noor MN, et al. Seed size and the evolution of leaf defences. J Ecol. 2015; 103: 1057–1068. doi: 10.1111/1365-2745.12407 | 0 | 1 |
| Kumar A, Bajpai O, Mishra AK, Sahu N, Behera SK, Bargali SS, et al. A checklist of the flowering plants of Katerniaghat Wildlife Sanctuary, Uttar Pradesh, India. J. Threat. Taxa. 2015; 7: 7309–7408 | 0 | 1 |
| Kuzmenkova SM. Plants of Belarus; 2015. Available: http://hbc.bas-net.by/plantae/eng/default.php. Accessed 15 February 2016 | 1 | 0 |
| Lazkov GA, Sultanova BA. Checklist of vascular plants of Kyrgyzstan. Helsinki: Botanical Museum, Finnish Museum of Natural History; 2011 | 1 | 0 |
| Le Houerou HN. Plant diversity in Marmarica (Libya & Egypt): a catalogue of the vascular plants reported with their biology, distribution, frequency, usage, economic potential, habitat and main ecological features, with an extensive bibliography. Candollea. 2004; 59: 259–308 | 1 | 1 |
| Letcher SG, Lasky JR, Chazdon RL, Norden N, Wright SJ, Meave JA, et al. Environmental gradients and the evolution of successional habitat specialization. A test case with 14 Neotropical forest sites. J Ecol. 2015; 103: 1276–1290. doi: 10.1111/1365-2745.12435 | 0 | 1 |
| Limbu D, Koirala M, Shang Z. A Checklist of Angiospermic Flora of Tinjure-Milke-Jaljale, Eastern Nepal. Nepal Journal of Science and Technology. 2013; 13: 87–96 | 0 | 1 |
| Linhart YB. Local biogeography of plants on a Caribbean atoll. Journal of Biogeography. 1980; 7: 159–171 | 0 | 1 |
| Lopez-Martinez JO, Sanaphre-Villanueva L, Dupuy JM, Hernandez-Stefanoni JL, Meave JA, Gallardo-Cruz JA. Beta-Diversity of functional groups of woody plants in a tropical dry forest in Yucatan. PLoS One. 2013; 8: e73660. doi: 10.1371/journal.pone.0073660 | 0 | 1 |
| Luke Q. Annotated Checklist of the Plants of the Shimba Hills, Kwale District, Kenya. Journal of East African Natural History. 2005; 94: 5–120 | 1 | 1 |
| Marticorena C, Squeo FA, Arancio G, Muñoz M. Catálogo de la flora vascular de la IV Región de Coquimbo. In: Squeo FA, Arancio G, Gutiérrez JR, editors. Libro rojo de la flora nativa y de los sitios prioritarios para su conservación: Región de Atacama: Ediciones Universidad de La Serena La Serena; 2008. pp. 105–142 | 1 | 1 |
| Marticorena C, Stuessy TF, Baeza CM. Catalogue of the vascular flora of the Robinson Crusoe or Juan Fernández islands, Chile. Gayana Botánica. 1998; 55: 187–211 | 0 | 1 |
| Masharabu T. Flore et végétation du Parc National de la Ruvubu au Burundi: diversité, structure et implications pour la conservation | 1 | 1 |
| Medina R, Reina-E M, Herrera E, Ávila FA, Chaparro O, Cortés-B. R. Catálogo preliminar da flora vascular dos bosques subandinos da cuchilla El Fara (Santander-Colômbia). Colombia Forestal. 2010; 13: 55–85 | 0 | 1 |
| Medjahdi B, Ibn Tattou M, Barkat D, Benabedli K. La flore vasculaire des Monts des Trara (Nord Ouest Algérien). Acta Botanica Malacitana. 2009; 34: 57–75 | 1 | 1 |
| Memariani F, Joharchi MR, Ejtehadi H, Emadzadeh K. Contributions to the flora and vegetation of Binalood Mountain range, NE Iran: Floristic and chorological studies in Fereizi region. Ferdowsi University International Journal of Biological Sciences. 2009; 1: 1–17 | 1 | 1 |
| Nikolić T. Flora Croatica Database; 2016. Available: http://hirc.botanic.hr/fcd. Accessed 12 February 2016 | 1 | 0 |
| Norton J, Majid SA, Allan D, Al Safran M, Böer B, Richer RA. An illustrated checklist of the flora of Qatar. Gosport, UK: Browndown Publications Gosport; 2009 | 1 | 1 |
| Notov AA. National park “Zavidovo”: vascular plants, bryophyte, lichens. Moscow; 2010 | 1 | 0 |
| NPS. NPSpecies. Information on Species in National Parks; 2015. Available: https://irma.nps.gov/NPSpecies/. Accessed 9 April 2015 | 1 | 0 |
| Oliveira-Filho AT. NeoTropTree. Flora arbórea da Região Neotropical: Um banco de dados envolvendo biogeografia, diversidade e conservação; 2014. Available: http://www.icb.ufmg.br/treeatlan/. Accessed 12 February 2016 | 0 | 1 |
| Pal D, Kumar A, Dutt B. Floristic diversity of Theog Forest Division, Himachal Pradesh, Western Himalaya. Check list. 2014; 10: 1083–1103 | 1 | 1 |
| Pandža M, Milović M, Kripina, Tafra D. Vascular flora of the Vrgada islets (Zadar Archipelago, Eastern Adriatic). Nat Croat. 2011; 20: 97–116 | 0 | 1 |
| Pandža M, Milović M. Flora of the islets near Pakoštane (Dalmatia, Croatia). Nat Croat. 2015; 24: 19–35. doi: 10.20302/NC.2015.24.2 | 0 | 1 |
| Pandža M, Milović M. Flora of the Veliki Lagan and Mali Lagan islets (Dugi Otok island, Croatia). Nat Croat. 2015; 24: 215–222. doi: 10.20302/NC.2015.24.13 | 0 | 1 |
| Pandža M, Skvorc Z. The flora of some uninhabited Sibenik Archipelago islands (Dalmatia, Croatia). Nat Croat. 2002; 11: 367–385 | 0 | 1 |
| Pandža M. Flora of the island of Zirje and the small islands around it (eastern Adriatic coast, Croatia). Acta Botica Croatia. 2003; 62: 115–139 | 0 | 1 |
| Pandža M. Flora of the small islands of Murter. Nat Croat. 2002; 11: 77–101 | 0 | 1 |
| Pandža M. Flora parka prirode Papuk (Slavonija, Hrvatska). Šumarski list. 2010; 134: 25–43 | 0 | 1 |
| Paula S, Arianoutsou M, Kazanis D, Tavsanoglu Ç, Lloret F, Buhk C, et al. Fire‐related traits for plant species of the Mediterranean Basin. Ecology. 2009; 90: 1420 | 0 | 1 |
| Pawlaczyk J, Pawlaczyk P. Vascular plants of Drawa National park and its neighbourhood; 2000. Available: http://www.eko.org.pl/lkp/dpn/chckl_rosliny_sh.html. Accessed 23 April 2015 | 1 | 0 |
| Pedashenko H, Vassilev K. Flora of Ponor Special Protection Area (Natura 2000), western Bulgaria. Acta zoologica bulgarica. 2014; 5: 33–60 | 1 | 1 |
| Peña-Chocarro MdC. Updated checklist of vascular plants of the Mbaracayú Forest Nature Reserve (Reserva Natural del Bosque Mbaracayú), Paraguay. Auckland, N.Z.: Magnolia Press; 2010 | 0 | 1 |
| Queensland Government. Census of the Queensland flora 2014; 2014. Available: https://data.qld.gov.au/dataset/census-of-the-queensland-flora-2014. Accessed 5 February 2015 | 1 | 0 |
| Rahman AHMM. Angiospermic Flora of Rajshahi District, Bangladesh. AJLS. 2013; 1: 105 | 0 | 1 |
| Rahman MS, Hossain GM, Khan SA, Uddin SN. An annotated Checklist of the Vascular Plants of Sundarban Mangrove Forest of Bangladesh. Bangladesh Journal of Plant Taxonomy. 2015; 22: 17–41 | 1 | 1 |
| Rakov NS, Saksonov S.V., Senator S.А., Vasjukov V.M. Vascular plants of Ulyanovsk Region. Togliatti: Russian Academy of Sciences; 2014 | 1 | 0 |
| Rossetto EFS, Vieira AOS. Vascular Flora of the Mata dos Godoy State Park, Londrina, Paraná, Brazil. Check list. 2013; 9: 1020–1034 | 0 | 1 |
| Royal Botanic Gardens and Domain Trust. PlantNET - The NSW Plant Information Network System; 2017. Available: http://plantnet.rbgsyd.nsw.gov.au. Accessed 16 September 2016 | 1 | 1 |
| Royal Botanic Gardens Kew. Seed information database (SID). v7.1; 2016. Available: http://data.kew.org/sid/. Accessed 22 March 2013 | 0 | 1 |
| Rundel PW, Dillon MO, Palma B. Flora and Vegetation of Pan de Azúcur National Park in the Atacama desert of Northern Chile. Gayana Bot. 1996; 53: 295–315 | 0 | 1 |
| SANBI. Plants of Southern Africa. An online checklist; 2014. Available: http://posa.sanbi.org. Accessed 9 March 2015 | 1 | 1 |
| Schaefer H, Hardy OJ, Silva L, Barraclough TG, Savolainen V. Testing Darwin's naturalization hypothesis in the Azores. Ecol Lett. 2011; 14: 389–396. doi: 10.1111/j.1461-0248.2011.01600.x | 0 | 1 |
| Schönfelder P, Schönfelder I. Die Kosmos-Kanarenflora. Über 850 Arten der Kanarenflora und 48 tropische Ziergehölze. Stuttgart: Franckh-Kosmos; 1997 | 0 | 1 |
| Scouppe M. Composition floristique et diversité de la végétation de la zone Est du Parc National de Taï (Côte d’Ivoire). Master, Université de Genève. 2011 | 0 | 1 |
| Selvi F. A critical checklist of the vascular flora of Tuscan Maremma (Grosseto province, Italy). Fl. Medit. 2010; 20: 47–139 | 0 | 1 |
| Senterre B, Chew MY, Chung RCK. Flora and vegetation of Pulau Babi Tengah, Johor, Peninsular Malaysia. Check list. 2015; 11: 1714. doi: 10.15560/11.4.1714 | 0 | 1 |
| Seregin AP. New Flora of the Maeshchera National Park (Vladimir Oblast, Russia). Checklist, distribution atlas, peculiarities, and distributional changes in species over the last decade (2002–2012). Tula: ASTRA; 2013 | 1 | 0 |
| Shaheen H, Qureshi R, Akram A, Gulfraz M, Potter D. A preliminary floristic checklist of Thal Desert Punjab, Pakistan. Pakistan Journal of Botany. 2014; 46: 13–18 | 1 | 0 |
| Shherbina S. Flora of Vascular Plants of Central Siberian State Biosperic Reserve and neighboring territories. Turczaninowia. 2009; 12: 71–241 | 1 | 0 |
| Silaeva TB, Chugunov GG, Kiryukhin IV, Ageeva AM, Vargot EV, Grishutkina GA, et al. Flora of the national park "Smolny". Mosses and vascular plants: annotated list of species [In Russian]: Commission of RAS for the Conservation of Biological Diversity [Комиссия РАН по сохранению биологического разнообразия] | 1 | 0 |
| Singh A. Observations on the vascular flora of Banaras Hindu University Main Campus, India. International Journal of Modern Biology and Medicine. 2015; 6: 48–87 | 1 | 1 |
| Skelin M, Ljubičić I, Skelin I, Vitasović Kosić I, Bogdanović S. The flora of Zečevo (Hvar Archipelago, Croatia). Agriculturae Conspectus Scientificus. 2014; 79: 85–91 | 0 | 1 |
| Slik FJW, Arroyo-Rodríguez V, Aiba S-I, Alvarez-Loayza P, Alves LF, Ashton P, et al. An estimate of the number of tropical tree species. Proceedings of the National Academy of Sciences of the United States of America. 2015; 112: 7472–7477. doi: 10.1073/pnas.1423147112 | 0 | 1 |
| SLUFG. Rote Liste und Artenliste Sachsen. Farn- und Samenpflanzen. Dresden: Sächsisches Landesamt für Umwelt, Landwirtschaft und Geologie; 2015 | 1 | 0 |
| Smith AC. Flora Vitiensis nova. a new Flora of Fiji (spermatophytes only): Pacific Tropical Botanical Garden (Lawaii, Hawaii); 1979-1996 | 0 | 1 |
| Smithsonian Institution. A checklist of Trees, Shrubs, Herbs, and Climbers of Myanmar. Contributions from the United States National Herbarium. 2003; 45: 1–590 | 1 | 0 |
| Stalmans M. Tinley's plant species list for the Greater Gorongosa ecosystem, Moçambique. Unpublished report by International Conservation Services to the Carr Foundation and the Ministry of Tourism; 2006 | 0 | 1 |
| Tamis WLM, van der Meijden R, Runhaar J, Bekker RM, Ozinga WA, Odé B, et al. Standard List of the Flora of the Netherlands 2003. Gorteria. 2004; 30: 101–195 | 1 | 1 |
| Ter Steege H, Vaessen RW, Cardenas-Lopez D, Sabatier D, Antonelli A, Oliveira SM de, et al. The discovery of the Amazonian tree flora with an updated checklist of all known tree taxa. Sci Rep. 2016; 6: 29549. doi: 10.1038/srep29549 | 0 | 1 |
| Thiombiano A, Schmidt M, Dressler S, Ouédraogo A, Hahn K. Catalogue des plantes vasculaires du Burkina Faso. Boissiera: mémoires des Conservatoire et Jardin botaniques de la Ville de Genève. 2012; 65: 1–391 | 1 | 1 |
| Tonkov S, Pavlova D, Atanassova J, Nedelcheva A, Marinova E. Floristis catalogue of the nature reserve Rilomanastirska Gora (Central Rila Mountains). I. The locality Kirilova: University of Sofia; 2004 | 1 | 1 |
| Tropicos. Catálogo de las Plantas Vasculares de Bolivia. Tropicos. St. Louis: Missouri Botanical Garden; 2015 | 1 | 1 |
| Tropicos. Catalogue of the Vascular Plants of Ecuador; 2015. Available: http://www.tropicos.org/Project/CE. Accessed 22 October 2015 | 1 | 1 |
| Tropicos. Catalogue of the Vascular Plants of the Department of Antioquia (Colombia). Tropicos. St. Louis: Missouri Botanical Garden; 2015 | 1 | 1 |
| Tropicos. Panama Checklist. Tropicos. St. Louis: Missouri Botanical Garden; 2015 | 1 | 1 |
| Tropicos. Paraguay Checklist. Tropicos. St. Louis: Missouri Botanical Garden; 2015 | 0 | 1 |
| Tropicos. Peru Checklist. Tropicos. St. Louis: Missouri Botanical Garden; 2015 | 1 | 1 |
| Turner IM. A catalogue of the vascular plants of Malaya. Singapore: Gardens' Bulletin; 1995 | 0 | 1 |
| Tutul E, Uddin MZ, Rahman MO, Hassan MA. Angiospermic flora of Runctia sal forest, Bangladesh. I. Liliopsida (Monocots). Bangladesh Journal of Plant Taxonomy. 2009; 16: 83–90 | 0 | 1 |
| UIB. Herbario virtual del Mediterráneo Occidental; 2007. Available: http://herbarivirtual.uib.es/cas-med/. Accessed 7 August 2012 | 0 | 1 |
| University of Greifswald. FloraGREIF - Virtual Flora of Mongolia; 2010. Available: http://greif.uni-greifswald.de/floragreif/. Accessed 2 February 2016 | 0 | 1 |
| USDA, NRCS. The PLANTS Database; 2015. Available: http://plants.usda.gov. Accessed 24 April 2015 | 1 | 1 |
| van Vreeswyk AME, Payne AL, Leighton KA, Hennig P. An inventory and condition survey of the Pilbara region, Western Australia: Department of Agriculture; 2004 | 0 | 1 |
| Vanderplank SE. The Vascular Flora of Greater San Quintín, Baja California, Mexico. CGU Theses & Dissertations; 2010 | 0 | 1 |
| Veklich TN. Flora of the Norsky Nature Reserve (Amur region). Blagoveshensk: Russian Academy of Sciences, Far East Division; 2009 | 1 | 0 |
| Velarde E, Wilder BT, Felcer RS, Ezcurra E. Floristic diversity and dynamics of Isla Rasa, Gulf of California - A globally important seabird island. Botanical Sciences. 2014; 92: 89–101 | 0 | 1 |
| Velarde EP, Guzmán RC, Koch SD. Plantas vasculares y vegetación de la parte alta del Arroyo Agua Fría, municipio de Minatitlán, Colima, México. Acta Botanica Mexicana. 2008; 84: 25–72 | 0 | 1 |
| VicFlora. Flora of Victoria; 2016. Available: http://vicflora.rbg.vic.gov.au. Accessed 30 November 2016 | 1 | 1 |
| Viciani D, Gonnelli V, Sirotti M, Agostini N. An annotated check-list of the vascular flora of the “Parco Nazionale delle Foreste Casentinesi, Monte Falterona e Campigna”(Northern Apennines Central Italy). Webbia. 2010; 65: 3–131 | 0 | 1 |
| Vogt C. Composición de la Flora Vascular del Chaco Boreal, Paraguay. I. Pteridophyta y Monocotiledoneae. Steviana. 2011; 3: 13–47 | 1 | 1 |
| Wagner WL, Herbst DR, Lorence DH. Flora of the Hawaiian Islands website; 2005. Available: http://botany.si.edu/pacificislandbiodiversity/hawaiianflora/. Accessed 16 October 2010 | 0 | 1 |
| WCSP. World Checklist of Selected Plant Families; 2014. Available: http://apps.kew.org/wcsp/home.do. Accessed 1 December 2014 | 1 | 1 |
| Webster GL, Rhode RM. Plant diversity of an Andean cloud forest: inventory of the vascular plants of Maquipucuna, Ecuador. Publications in Botany. 2001; 82: 1–228 | 0 | 1 |
| Western Australian Herbarium. FloraBase - the Western Australian Flora; 2017. Available: https://florabase.dpaw.wa.gov.au/. Accessed 16 September 2016 | 1 | 1 |
| Wieringa JJ. Flora of Gabon. unpublished; 23.04.2016 | 0 | 1 |
| Woodroffe CD. Vascular plant speciesarea relationships on Nui Atoll, Tuvalu, Central Pacific: a reassessment of the small island effect. Australien Journal of Ecology. 1986; 11: 21–31 | 0 | 1 |
| Yineger H, Kelbessa E, Bekele T, Lulekal E. Floristic composition and structure of the dry afromontane forest at Bale Mountains National Park, Ethiopia. SINET: Ethiopian Journal of Science. 2008; 31: 103–120 | 1 | 1 |
| ZDSF, SKEW. Info Flora. Artenliste Schweiz 5x5 km; 2014. Available: https://www.infoflora.ch/de/daten-beziehen/artenliste-5x5-km.html. Accessed 13 February 2015 | 1 | 0 |
| Zhang ST, Zhen Du G, Chen JK. Seed size in relation to phylogeny, growth form and longevity in a subalpine meadow on the east of the Tibetan plateau. Folia Geobot. 2004; 39: 129–142. doi: 10.1007/BF02805242 | 0 | 1 |
| Zuloaga FO, Morrone O, Belgrano M. Catálogo de las Plantas Vasculares del Cono Sur; 2014. Available: http://www.darwin.edu.ar/Proyectos/FloraArgentina/fa.htm. Accessed 16 March 2015 | 1 | 1 |
| Zvyagintseva KO. An annotated checklist of the urban flora of Kharkiv. Kharkiv, Ukraine: Kharkiv National University; 2015 | 1 | 1 |
| Абрамова ЛА, Волкова ПА. Сосудистые растения Байкальского заповедника. (Аннотированный список видов). Флора и фауна заповедников. 2011; 117: 1–112 | 1 | 0 |
| Антипова EM. Флора внутриконтинентальных островных лесостепей Средней Сибири. Красноярск: гос. пед. ун-т им. В.П. Астафьева; 2012 | 1 | 0 |
| Артемов ИА. Определитель растений Катунского биосферного заповедника. БАРНАУЛ: Russian Academy of Sciences; 2012 | 1 | 1 |
| Гаджиев ВД, Юсифов ЭФ. Флора и растительность Кызылагачского заповедника и их биоразнообразие. Баку: Национальная Академия Наук Азербайджана; 2003 | 1 | 0 |
| Евстигнеев ОИ, Федотов ЮП. Флора сосудистых растений заповедника "Брянский лес". Брянск: Гос. природ. биосфер. заповедник Брян. лес; 2007 | 1 | 0 |
| Куликов ПВ, Кирсанова ОФ. Сосудистые растения заповедника "Денежкин камень". (Аннотированный список видов). Флора и фауна заповедников. 2012; 119: 1–140 | 1 | 0 |
| Лактионов АП, Пилипенко ВН, Глаголев СБ, Лактионова НА. Сосудистые растения заповедника «Богдинско-Баскунчакский» (Аннотированный список видов): Флора и фауна заповедников. Флора и фауна заповедников. 2008; 113: 1–113 | 1 | 0 |
| Миркина БМ. Флора и растительность Национального парка «Башкирия»: Russian Academy of Sciences; 2010 | 1 | 0 |
| Морозова ОВ, Царевская НГ, Белоновская ЕА. Сосудистые растения национального парка «Валдайский». Флора и фауна заповедников. 2010; 7: 1–98 | 1 | 0 |
| Хапугин AA. Сосудистые растения Ромодановского района Республики Мордовия (конспект флоры). Saransk; 2013 | 1 | 0 |

**References**

1. Conti F, Abbate G, Alessandrini A, Blasi C. Annotated Checklist of the Italian Vascular Flora. Italy: Palombi Editori, Roma; 2005.

2. Abderrahman A, El Kadmiri AA. Richesse et diversité floristique de la subéraie de la Mamora (Maroc). Acta Botanica Malacitana. 2005; 30: 127–138.

3. Badshah L, Hussain F, Sher Z. Floristic inventory, ecological characteristics and biological spectrum of rangeland, District Tank, Pakistan. Pak. J. Bot. 2013; 45: 1159–1168.

4. Barker WR, Barker RM, Jessop JP, Vonow HP. Census of South Australian vascular plants. Journal of the Adelaide Botanic Gardens Supplement. 2005; 1: 1–396.

5. BioScripts. Flora Vascular; 2014. Available: http://www.floravascular.com/. Accessed 25 May 2014.

6. Bridgewater SGM, Harris DJ, Whitefoord C, Monro AK, Penn MG, Sutton DA, et al. A Preliminary Checklist of the vascular plants of the Chiquibul Forest, Belize. Edin. Jnl of Bot. 2006; 63: 269–321.

7. Brundu G, Camarda I. The Flora of Chad: a checklist and brief analysis. Phytokeys. 2013; 23: 1–17.

8. Chawla A, Parkash O, Sharma V, Rajkumar S, Lal B, Gopichand, et al. Vascular plants, Kinnaur, Himachal Pradesh, India. Check list. 2012; 8: 321–348.

9. Doroftei M, Oprea A, Ştefan N, Sârbu I. Vascular wild flora of Danube Delta Biosphere Reserve. Sci. Annals of Danube Delta Institute. 2011; 17: 15–52.

10. Egea J de, Peña-Chocarro M, Espada C, Knapp S. Checklist of vascular plants of the Department of Ñeembucú, Paraguay. Phytokeys. 2012; 9: 15–179.

11. Ejaz-ul-Islam Dar M, Cochard R, Shrestha RP, Ahmad S. Floristic composition of Machiara National Park, District Muzaffarabad Azad Kashmir, Pakistan. International Journal of Biosciences. 2012; 2: 28–45.

12. El-Ghani MM, Abdel-Khalik KN. Floristic Diversity and Phytogeography of the Gebel Elba National Park, South-East Egypt. Turkish Journal of Botany. 2005; 30: 121–136.

13. Fischer E, Rembold K, Althof A, Obholzer J, Malombe I, Mwachala G, et al. Annotated checklist of the vascular plants of Kakamega Forest, Western Province, Kenya. Journal of East African Natural History. 2010; 99: 129–226.

14. Funk VA, Hollowell T, Berry P, Kelloff C, Alexander SN. Checklist of the plants of the Guiana Shield (Venezuela: Amazonas, Bolivar, Delta Amacuro; Guyana, Surinam, French Guiana). Washington, DC: Department of Botany, National Museum of Natural History; 2007.

15. ZDSF, SKEW. Info Flora. Artenliste Schweiz 5x5 km; 2014. Available: https://www.infoflora.ch/de/daten-beziehen/artenliste-5x5-km.html. Accessed 13 February 2015.

16. Zuloaga FO, Morrone O, Belgrano M. Catálogo de las Plantas Vasculares del Cono Sur; 2014. Available: http://www.darwin.edu.ar/Proyectos/FloraArgentina/fa.htm. Accessed 16 March 2015.

17. Klimeš L, Dickoré B. Flora of Ladak (NW Himalaya). A preliminary check-list; 2015. Available: http://www.butbn.cas.cz/klimes/desert.html. Accessed 12 January 2015.

18. Le Houerou HN. Plant diversity in Marmarica (Libya & Egypt): a catalogue of the vascular plants reported with their biology, distribution, frequency, usage, economic potential, habitat and main ecological features, with an extensive bibliography. Candollea. 2004; 59: 259–308.

19. Luke Q. Annotated Checklist of the Plants of the Shimba Hills, Kwale District, Kenya. Journal of East African Natural History. 2005; 94: 5–120.

20. Marticorena C, Squeo FA, Arancio G, Muñoz M. Catálogo de la flora vascular de la IV Región de Coquimbo. In: Squeo FA, Arancio G, Gutiérrez JR, editors. Libro rojo de la flora nativa y de los sitios prioritarios para su conservación: Región de Atacama: Ediciones Universidad de La Serena La Serena; 2008. pp. 105–142.

21. Memariani F, Joharchi MR, Ejtehadi H, Emadzadeh K. Contributions to the flora and vegetation of Binalood Mountain range, NE Iran: Floristic and chorological studies in Fereizi region. Ferdowsi University International Journal of Biological Sciences. 2009; 1: 1–17.

22. Norton J, Majid SA, Allan D, Al Safran M, Böer B, Richer RA. An illustrated checklist of the flora of Qatar. Gosport, UK: Browndown Publications Gosport; 2009.

23. Pal D, Kumar A, Dutt B. Floristic diversity of Theog Forest Division, Himachal Pradesh, Western Himalaya. Check list. 2014; 10: 1083–1103.

24. Queensland Government. Census of the Queensland flora 2014; 2014. Available: https://data.qld.gov.au/dataset/census-of-the-queensland-flora-2014. Accessed 5 February 2015.

25. SLUFG. Rote Liste und Artenliste Sachsen. Farn- und Samenpflanzen. Dresden: Sächsisches Landesamt für Umwelt, Landwirtschaft und Geologie; 2015.

26. SANBI. Plants of Southern Africa. An online checklist; 2014. Available: http://posa.sanbi.org. Accessed 9 March 2015.

27. Seregin AP. New Flora of the Maeshchera National Park (Vladimir Oblast, Russia). Checklist, distribution atlas, peculiarities, and distributional changes in species over the last decade (2002–2012). Tula: ASTRA; 2013.

28. Shaheen H, Qureshi R, Akram A, Gulfraz M, Potter D. A preliminary floristic checklist of Thal Desert Punjab, Pakistan. Pakistan Journal of Botany. 2014; 46: 13–18.

29. Smithsonian Institution. A checklist of Trees, Shrubs, Herbs, and Climbers of Myanmar. Contributions from the United States National Herbarium. 2003; 45: 1–590.

30. USDA, NRCS. The PLANTS Database; 2015. Available: http://plants.usda.gov. Accessed 24 April 2015.

31. Vogt C. Composición de la Flora Vascular del Chaco Boreal, Paraguay. I. Pteridophyta y Monocotiledoneae. Steviana. 2011; 3: 13–47.

32. Bakis Y, Babac MT, Uslu E. Tübives. Turkish Plants Data Service; 2015. Available: http://www.tubives.com/. Accessed 13 February 2015.

33. Dauby G, Leal M, Stevart T. Vascular plant checklist of the coastal National Park of Pongara, Gabon. Systematics and geography of plants. 2008; 78: 155–216.

34. Pawlaczyk J, Pawlaczyk P. Vascular plants of Drawa National park and its neighbourhood; 2000. Available: http://www.eko.org.pl/lkp/dpn/chckl_rosliny_sh.html. Accessed 23 April 2015.

35. Thiombiano A, Schmidt M, Dressler S, Ouédraogo A, Hahn K. Catalogue des plantes vasculaires du Burkina Faso. Boissiera: mémoires des Conservatoire et Jardin botaniques de la Ville de Genève. 2012; 65: 1–391.

36. Лактионов АП, Пилипенко ВН, Глаголев СБ, Лактионова НА. Сосудистые растения заповедника «Богдинско-Баскунчакский» (Аннотированный список видов): Флора и фауна заповедников. Флора и фауна заповедников. 2008; 113: 1–113.

37. Куликов ПВ, Кирсанова ОФ. Сосудистые растения заповедника "Денежкин камень". (Аннотированный список видов). Флора и фауна заповедников. 2012; 119: 1–140.

38. Гаджиев ВД, Юсифов ЭФ. Флора и растительность Кызылагачского заповедника и их биоразнообразие. Баку: Национальная Академия Наук Азербайджана; 2003.

39. Абрамова ЛА, Волкова ПА. Сосудистые растения Байкальского заповедника. (Аннотированный список видов). Флора и фауна заповедников. 2011; 117: 1–112.

40. Pedashenko H, Vassilev K. Flora of Ponor Special Protection Area (Natura 2000), western Bulgaria. Acta zoologica bulgarica. 2014; 5: 33–60.

41. Masharabu T. Flore et végétation du Parc National de la Ruvubu au Burundi: diversité, structure et implications pour la conservation.

42. Adou Yao CY. Diversité floristique et végétation dans le Parc National de Taï, Côte d'Ivoire. Abidjan: Tropenbos Côte d'Ivoire; 2005.

43. Hoang VS. Uses and conservation of plant diversity in Ben En National Park, Vietnam. Ph.D., National Herbarium of the Netherlands, Leiden University Branch. 2009.

44. Cuc Phuong National Park. Flora of the Cuc Phuong National Park. Vietnam; 2011.

45. NPS. NPSpecies. Information on Species in National Parks; 2015. Available: https://irma.nps.gov/NPSpecies/. Accessed 9 April 2015.

46. ICIMOD. Hindu Kush-Himalayan (HKH) Conservation Portal; 2015. Available: http://www.icimod.org/hkhconservationportal/Initiatives.aspx. Accessed 21 April 2015.

47. Yineger H, Kelbessa E, Bekele T, Lulekal E. Floristic composition and structure of the dry afromontane forest at Bale Mountains National Park, Ethiopia. SINET: Ethiopian Journal of Science. 2008; 31: 103–120.

48. WCSP. World Checklist of Selected Plant Families; 2014. Available: http://apps.kew.org/wcsp/home.do. Accessed 1 December 2014.

49. Морозова ОВ, Царевская НГ, Белоновская ЕА. Сосудистые растения национального парка «Валдайский». Флора и фауна заповедников. 2010; 7: 1–98.

50. Catarino L, Martins ES, Basto MF, Diniz MA. An annotated checklist of the vascular flora of Guinea-Bissau (West Africa). Blumea-Biodiversity, Evolution and Biogeography of Plants. 2008; 53: 1–222.

51. Rahman MS, Hossain GM, Khan SA, Uddin SN. An annotated Checklist of the Vascular Plants of Sundarban Mangrove Forest of Bangladesh. Bangladesh Journal of Plant Taxonomy. 2015; 22: 17–41.

52. Shherbina S. Flora of Vascular Plants of Central Siberian State Biosperic Reserve and neighboring territories. Turczaninowia. 2009; 12: 71–241.

53. Kabuye CHS, Mungai GM, Mutangah JG. Flora of Kora National Reserve. In: Coe M, Collins NM, editors. Kora: An Ecological Inventory of the Kora National Reserve, Kenya. Kora Research Project 1982-85 : a Joint Venture Between the National Museums of Kenya and the Royal Geographical Society: Royal Geographical Society; 1986. pp. 57–104.

54. Veklich TN. Flora of the Norsky Nature Reserve (Amur region). Blagoveshensk: Russian Academy of Sciences, Far East Division; 2009.

55. Azbukina ZM, Bardunov LV, Bezdeleva TA, Bogacheva AV, Bulakh EM, Vasilyeva LN, et al. Flora, vegetation and mycobiota of the reserve «Ussuriysky». Vladivostok: Dalnauka; 2006.

56. Евстигнеев ОИ, Федотов ЮП. Флора сосудистых растений заповедника "Брянский лес". Брянск: Гос. природ. биосфер. заповедник Брян. лес; 2007.

57. Evarts-Bunders P, Evarte-Bundere G, Bāra J, Nitcis M. The flora of vascular plants in nature reserve „Eglone”. Acta Biologica Universitatis Daugavpiliensis. 2013; 13: 21–38.

58. Grozeva NH. The flora of Atanasovsko lake natural reserve. In: Gruev B, Nikolova M, Donev A, editors. Proceedings of the Balkan Scientific Conference of Biology; 2005. pp. 381–396.

59. Lazkov GA, Sultanova BA. Checklist of vascular plants of Kyrgyzstan. Helsinki: Botanical Museum, Finnish Museum of Natural History; 2011.

60. Tonkov S, Pavlova D, Atanassova J, Nedelcheva A, Marinova E. Floristis catalogue of the nature reserve Rilomanastirska Gora (Central Rila Mountains). I. The locality Kirilova: University of Sofia; 2004.

61. Tropicos. Catálogo de las Plantas Vasculares de Bolivia. Tropicos. St. Louis: Missouri Botanical Garden; 2015.

62. Tropicos. Catalogue of the Vascular Plants of the Department of Antioquia (Colombia). Tropicos. St. Louis: Missouri Botanical Garden; 2015.

63. Tropicos. Catalogue of the Vascular Plants of Ecuador; 2015. Available: http://www.tropicos.org/Project/CE. Accessed 22 October 2015.

64. Tropicos. Panama Checklist. Tropicos. St. Louis: Missouri Botanical Garden; 2015.

65. Tropicos. Peru Checklist. Tropicos. St. Louis: Missouri Botanical Garden; 2015.

66. Артемов ИА. Определитель растений Катунского биосферного заповедника. БАРНАУЛ: Russian Academy of Sciences; 2012.

67. Миркина БМ. Флора и растительность Национального парка «Башкирия»: Russian Academy of Sciences; 2010.

68. Gafurova ММ. Vascular plants of Chuvash Republic. Flora of the Volga River Basin V. III: The Russian Academy of Sciences; 2014.

69. Zvyagintseva KO. An annotated checklist of the urban flora of Kharkiv. Kharkiv, Ukraine: Kharkiv National University; 2015.

70. Notov AA. National park “Zavidovo”: vascular plants, bryophyte, lichens. Moscow; 2010.

71. Хапугин AA. Сосудистые растения Ромодановского района Республики Мордовия (конспект флоры). Saransk; 2013.

72. Rakov NS, Saksonov S.V., Senator S.А., Vasjukov V.M. Vascular plants of Ulyanovsk Region. Togliatti: Russian Academy of Sciences; 2014.

73. Антипова EM. Флора внутриконтинентальных островных лесостепей Средней Сибири. Красноярск: гос. пед. ун-т им. В.П. Астафьева; 2012.

74. Botanical Garden Tel Aviv. Israel Flora: Tel Aviv University.

75. Medjahdi B, Ibn Tattou M, Barkat D, Benabedli K. La flore vasculaire des Monts des Trara (Nord Ouest Algérien). Acta Botanica Malacitana. 2009; 34: 57–75.

76. Chinese Virtual Herbarium. The Flora of China v. 5.0; 2016. Available: http://www.cvh.org.cn/. Accessed 15 January 2016.

77. Bernal R, Gradstein SR, Celis M. Catálogo de plantas y líquenes de Colombia; 2015. Available: http://catalogoplantasdecolombia.unal.edu.co/. Accessed 15 January 2016.

78. Jardim Botânico do Rio de Janeiro. Flora do Brasil 2020 em construção; 2016. Available: http://floradobrasil.jbrj.gov.br/. Accessed 9 May 2016.

79. Danihelka J, Chrtek J, Kaplan Z. Checklist of vascular plants of the Czech Republic. Preslia. 2012; 84: 647–811.

80. Nikolić T. Flora Croatica Database; 2016. Available: http://hirc.botanic.hr/fcd. Accessed 12 February 2016.

81. Kuzmenkova SM. Plants of Belarus; 2015. Available: http://hbc.bas-net.by/plantae/eng/default.php. Accessed 15 February 2016.

82. Bancheva S, Vassilev K. Vascular flora of the Beli Lom Nature Reserve in Northeast Bulgaria. Phytologia Balcanica. 2006; 12: 377–386.

83. Singh A. Observations on the vascular flora of Banaras Hindu University Main Campus, India. International Journal of Modern Biology and Medicine. 2015; 6: 48–87.

84. Silaeva TB, Chugunov GG, Kiryukhin IV, Ageeva AM, Vargot EV, Grishutkina GA, et al. Flora of the national park "Smolny". Mosses and vascular plants: annotated list of species [In Russian]: Commission of RAS for the Conservation of Biological Diversity [Комиссия РАН по сохранению биологического разнообразия].

85. Dimopoulos P, Raus T, Bergmeier E, Constantinidis T, Iatrou G, Kokkini S, et al. Vascular plants of Greece. An annotated checklist. Berlin, Athens: Botanischer Garten und Botanisches Museum Berlin-Dahlem, Freie Universität Berlin; Hellenic Botanical Society; 2013.

86. Bundesamt für Naturschutz. Floraweb; 2016. Available: www.floraweb.de. Accessed 15 September 2016.

87. Cochard R, Bloesch U. Electronic plant species database of the Saadani National Park, coastal Tanzania; 2007. Available: http://www.wildlife-baldus.com/saadani.html. Accessed 14 November 2016.

88. Hyde MA, Wursten BT, Ballings P, Coates Palgrave M. Flora of Botswana; 2016. Available: http://www.botswanaflora.com/. Accessed 14 November 2016.

89. Bingham MG, Willemen A, Wursten BT, Ballings P, Hyde MA. Flora of Zambia; 2016. Available: http://www.zambiaflora.com/. Accessed 14 November 2016.

90. Hyde MA, Wursten BT, Ballings P, Coates Palgrave M. Flora of Zimbabwe; 2016. Accessed 14 November 2016.

91. Hyde MA, Wursten BT, Ballings P, Coates Palgrave M. Flora of Mozambique; 2016. Available: http://www.mozambiqueflora.com/. Accessed 14 November 2016.

92. Hyde MA, Wursten BT, Ballings P, Coates Palgrave M. Flora of Malawi; 2016. Available: http://www.malawiflora.com/index.php. Accessed 14 November 2016.

93. Fischer MA, Adler W, Oswald K. Exkursionsflora für Österreich, Liechtenstein und Südtirol. Bestimmungsbuch für alle in der Republik Österreich, im Fürstentum Liechtenstein und in der Autonomen Provinz Bozen. 3rd ed. Linz: OÖ Landesmuseum; 2008.

94. Chang C-S, Kim H, Chang K. Provisional Checklist of the Vascular Plants for the Korea Peninsular Flora (KPF). Version 1.0. Korea.

95. VicFlora. Flora of Victoria; 2016. Available: http://vicflora.rbg.vic.gov.au. Accessed 30 November 2016.

96. Dauby G, Zaiss R, Blach-Overgaard A, Catarino L, Damen T, Deblauwe V, et al. RAINBIO. A mega-database of tropical African vascular plants distributions. PK. 2016; 74: 1–18. doi: 10.3897/phytokeys.74.9723.

97. CONABIO. Sistema Nacional de Información sobre Biodiversidad; 2016. Available: https://www.gob.mx/conabio. Accessed 11 April 2016.

98. Tamis WLM, van der Meijden R, Runhaar J, Bekker RM, Ozinga WA, Odé B, et al. Standard List of the Flora of the Netherlands 2003. Gorteria. 2004; 30: 101–195.

99. Western Australian Herbarium. FloraBase - the Western Australian Flora; 2017. Available: https://florabase.dpaw.wa.gov.au/. Accessed 16 September 2016.

100. Royal Botanic Gardens and Domain Trust. PlantNET - The NSW Plant Information Network System; 2017. Available: http://plantnet.rbgsyd.nsw.gov.au. Accessed 16 September 2016.

101. Breckle S-W, Hedge IC, Rafiqpoor MD. Vascular plants of Afghanistan. An augmented checklist. Bonn: Scientia Bonnensis; 2013.

102. Wagner WL, Herbst DR, Lorence DH. Flora of the Hawaiian Islands website; 2005. Available: http://botany.si.edu/pacificislandbiodiversity/hawaiianflora/. Accessed 16 October 2010.

103. Ferreira AL, Coutinho BR, Pinheiro HT, Thomaz LD. Composição florística e formações vegetais da Ilha dos Franceses, Espírito Santo. Bol. Mus. Biol. Mello Leitao. 2007; 22: 25–44.

104. Kemenes A. Distribuição espacial da flora terrestre fanerogâmica do Parque Nacional Marinho de Abrolhos, BA. Revista Brasil. Bot. 2003; 26: 141–150.

105. Chong KY, Tan TWH, Corlett RT. A checklist of the total vascular plant flora of Singapore. Native, Naturalised and Cultivated Species. Singapore: Raffles Museum of Biodiversity Research; 2009.

106. UIB. Herbario virtual del Mediterráneo Occidental; 2007. Available: http://herbarivirtual.uib.es/cas-med/. Accessed 7 August 2012.

107. Marticorena C, Stuessy TF, Baeza CM. Catalogue of the vascular flora of the Robinson Crusoe or Juan Fernández islands, Chile. Gayana Botánica. 1998; 55: 187–211.

108. Smith AC. Flora Vitiensis nova. a new Flora of Fiji (spermatophytes only): Pacific Tropical Botanical Garden (Lawaii, Hawaii); 1979-1996.

109. Velarde E, Wilder BT, Felcer RS, Ezcurra E. Floristic diversity and dynamics of Isla Rasa, Gulf of California - A globally important seabird island. Botanical Sciences. 2014; 92: 89–101.

110. Costion C, Lorence D. The endemic plants of Micronesia. A geographical checklist and commentary. Micronesica. 2012; 43: 51–100.

111. Keppel G. Summary report on forests of the Mataqali Nadicake Kilaka, Kubulau district, Bua, Vanua Levu; 2005.

112. Skelin M, Ljubičić I, Skelin I, Vitasović Kosić I, Bogdanović S. The flora of Zečevo (Hvar Archipelago, Croatia). Agriculturae Conspectus Scientificus. 2014; 79: 85–91.

113. 3D Environmental. Profile for management of the habitats and related ecological and cultural resource values of Boigu Island; 2013.

114. Figueiredo E, Paiva J, Stevart T, Oliveira F, Smith GF. Annotated catalogue of the flowering plants of São Tomé and Príncipe. Bothalia. 2011; 41: 41–82.

115. Senterre B, Chew MY, Chung RCK. Flora and vegetation of Pulau Babi Tengah, Johor, Peninsular Malaysia. Check list. 2015; 11: 1714. doi: 10.15560/11.4.1714.

116. Schönfelder P, Schönfelder I. Die Kosmos-Kanarenflora. Über 850 Arten der Kanarenflora und 48 tropische Ziergehölze. Stuttgart: Franckh-Kosmos; 1997.

117. Kim C-S, Koh J-G, Moon M-O, Song G-P, Hyun H-J, Song K-M, et al. Flora and life form spectrum of Hallasan natural reserve, Korea. Journal of the Environmental Sciences. 2007; 16: 1257–1269.

118. Woodroffe CD. Vascular plant speciesarea relationships on Nui Atoll, Tuvalu, Central Pacific: a reassessment of the small island effect. Australien Journal of Ecology. 1986; 11: 21–31.

119. Linhart YB. Local biogeography of plants on a Caribbean atoll. Journal of Biogeography. 1980; 7: 159–171.

120. Jasprica N, Dolina K, Milović M. Plant taxa and communities on three islets in south Croatia, NE Mediterranean. Nat Croat. 2015; 24: 191–213. doi: 10.20302/NC.2015.24.12.

121. Pandža M, Skvorc Z. The flora of some uninhabited Sibenik Archipelago islands (Dalmatia, Croatia). Nat Croat. 2002; 11: 367–385.

122. Pandža M. Flora of the small islands of Murter. Nat Croat. 2002; 11: 77–101.

123. Pandža M. Flora of the island of Zirje and the small islands around it (eastern Adriatic coast, Croatia). Acta Botica Croatia. 2003; 62: 115–139.

124. Pandža M, Milović M. Flora of the islets near Pakoštane (Dalmatia, Croatia). Nat Croat. 2015; 24: 19–35. doi: 10.20302/NC.2015.24.2.

125. Pandža M, Milović M, Kripina, Tafra D. Vascular flora of the Vrgada islets (Zadar Archipelago, Eastern Adriatic). Nat Croat. 2011; 20: 97–116.

126. Pandža M, Milović M. Flora of the Veliki Lagan and Mali Lagan islets (Dugi Otok island, Croatia). Nat Croat. 2015; 24: 215–222. doi: 10.20302/NC.2015.24.13.

127. Abdel Khalik K, El-Sheikh M, El-Aidarous A. Floristic diversity and vegetation analysis of Wadi Al-Noman, Mecca, Saudi Arabia. Turkish Journal of Botany. 2013; 37: 894–907.

128. Arakaki M, Cano A. Composición florística de la cuenca del río Ilo-Moquegua y Lomas de Ilo, Moquegua, Peru. Revista Peruana de Biología. 2003; 10: 5–19.

129. Brennan K. An annotated checklist of the vascular plants of the Alligator Rivers Region, Northern Territory, Australia. Barton, Australia: Supervising Scientist; 1996.

130. Clark JL, Neill DA, Asanza M. Floristic checklist of the Mache-Chindul mountains of Northwestern Ecuador. Contributions from the United States National Herbarium. 2006; 54: 1–180.

131. Desmet P, Brouillet L. Database of Vascular Plants of Canada (VASCAN): a community contributed taxonomic checklist of all vascular plants of Canada, Saint Pierre and Miquelon, and Greenland. Phytokeys. 2013; 25: 55–67.

132. Domínguez E, Marticorena C, Elvebakk A, Pauchard A. Catálogo de la flora vascular del Parque Nacional Pali Aike. XII Región, Chile. Gayana Botánica. 2004; 61: 67–72.

133. Espinosa-Jiménez JA, Pérez-Farrera MÁ, Martínez-Camilo R. Inventario florístico del Parque Nacional Cañón del Sumidero, Chiapas, México. Boletín de la Sociedad Botánica de México. 2011; 89: 37–82.

134. Figueroa-C. Y, Galeano G. Lista comentada de las plantas vasculares del enclave seco interandino de La Tatacoa (Huila, Colombia). Caldasia. 2007; 29: 263–281.

135. Harris DJ. The vascular plants of the Dzanga-Sangha Reserve, Central African Republic. Edinburgh, Scotland, UK: Royal Botanic Garden Edinburgh; 2002.

136. Medina R, Reina-E M, Herrera E, Ávila FA, Chaparro O, Cortés-B. R. Catálogo preliminar da flora vascular dos bosques subandinos da cuchilla El Fara (Santander-Colômbia). Colombia Forestal. 2010; 13: 55–85.

137. Peña-Chocarro MdC. Updated checklist of vascular plants of the Mbaracayú Forest Nature Reserve (Reserva Natural del Bosque Mbaracayú), Paraguay. Auckland, N.Z.: Magnolia Press; 2010.

138. Rundel PW, Dillon MO, Palma B. Flora and Vegetation of Pan de Azúcur National Park in the Atacama desert of Northern Chile. Gayana Bot. 1996; 53: 295–315.

139. Selvi F. A critical checklist of the vascular flora of Tuscan Maremma (Grosseto province, Italy). Fl. Medit. 2010; 20: 47–139.

140. Viciani D, Gonnelli V, Sirotti M, Agostini N. An annotated check-list of the vascular flora of the “Parco Nazionale delle Foreste Casentinesi, Monte Falterona e Campigna”(Northern Apennines Central Italy). Webbia. 2010; 65: 3–131.

141. Webster GL, Rhode RM. Plant diversity of an Andean cloud forest: inventory of the vascular plants of Maquipucuna, Ecuador. Publications in Botany. 2001; 82: 1–228.

142. Alves, Ruy José Válka, Kolbek J. Summit vascular flora of Serra de São José, Minas Gerais, Brazil. Check list. 2009; 5: 35–73.

143. Rossetto EFS, Vieira AOS. Vascular Flora of the Mata dos Godoy State Park, Londrina, Paraná, Brazil. Check list. 2013; 9: 1020–1034.

144. van Vreeswyk AME, Payne AL, Leighton KA, Hennig P. An inventory and condition survey of the Pilbara region, Western Australia: Department of Agriculture; 2004.

145. Stalmans M. Tinley's plant species list for the Greater Gorongosa ecosystem, Moçambique. Unpublished report by International Conservation Services to the Carr Foundation and the Ministry of Tourism; 2006.

146. Gnoumou A, Ouedraogo O, Schmidt M, Thiombiano A. Floristic diversity of classified forest and partial faunal reserve of Comoé-Léraba, southwest Burkina Faso. Check list. 2015; 11: 1557.

147. Tutul E, Uddin MZ, Rahman MO, Hassan MA. Angiospermic flora of Runctia sal forest, Bangladesh. I. Liliopsida (Monocots). Bangladesh Journal of Plant Taxonomy. 2009; 16: 83–90.

148. Cascante-Marín A, Estrada-Chavarría A. Las plantas vasculares de El Rodeo, Costa Rica. Brenesia. 2012; 77: 71–128.

149. Kraaij T. The flora of the Bontebok National Park in regional perspective. South African Journal of Botany. 2011; 77: 455–473. doi: 10.1016/j.sajb.2010.09.013.

150. Limbu D, Koirala M, Shang Z. A Checklist of Angiospermic Flora of Tinjure-Milke-Jaljale, Eastern Nepal. Nepal Journal of Science and Technology. 2013; 13: 87–96.

151. Assédé EPS, Adomou AC, Sinsin B. Magnoliophyta, Biosphere Reserve of Pendjari, Atacora Province, Benin. Check list. 2012; 8: 642–661.

152. Rahman AHMM. Angiospermic Flora of Rajshahi District, Bangladesh. AJLS. 2013; 1: 105.

153. de la Luz, León, Rebman J, Domínguez-León M, Domínguez-Cadena R. The vascular flora and floristic relationships of the sierra de la Giganta in Baja California Sur, México. Revista mexicana de biodiversidad. 2008; 79: 29–65.

154. Scouppe M. Composition floristique et diversité de la végétation de la zone Est du Parc National de Taï (Côte d’Ivoire). Master, Université de Genève. 2011.

155. Kleyer M, Bekker RM, Knevel IC, Bakker JP, Thompson K, Sonnenschein M, et al. The LEDA Traitbase: a database of life-history traits of the Northwest European flora. J Ecol. 2008; 96: 1266–1274.

156. Hawkins BA, Rueda M, Rangel TF, Field R, Diniz-Filho JAF, Linder P. Community phylogenetics at the biogeographical scale: cold tolerance, niche conservatism and the structure of North American forests. J. Biogeogr. 2013; 41: 23–38.

157. Hauenstein E, Muñoz-Pedreros A, Yánez J, Sánchez P, Möller P, Guiñez B, et al. Flora y vegetación de la Reserva Nacional Lago Peñuelas, Reserva de la Biósfera, Región de Valparaíso, Chile. Bosque (Valdivia). 2009; 30: 159–179. doi: 10.4067/S0717-92002009000300006.

158. Jordano P. FRUBASE; 2008. Available: http://ebd10.ebd.csic.es/mywork/frubase/frubase.html. Accessed 26.06.15.

159. Fern K, Fern A. Useful Tropical Plants Database; 2015. Accessed 25 July 2015.

160. Velarde EP, Guzmán RC, Koch SD. Plantas vasculares y vegetación de la parte alta del Arroyo Agua Fría, municipio de Minatitlán, Colima, México. Acta Botanica Mexicana. 2008; 84: 25–72.

161. Slik FJW, Arroyo-Rodríguez V, Aiba S-I, Alvarez-Loayza P, Alves LF, Ashton P, et al. An estimate of the number of tropical tree species. Proceedings of the National Academy of Sciences of the United States of America. 2015; 112: 7472–7477. doi: 10.1073/pnas.1423147112.

162. Kumar A, Bajpai O, Mishra AK, Sahu N, Behera SK, Bargali SS, et al. A checklist of the flowering plants of Katerniaghat Wildlife Sanctuary, Uttar Pradesh, India. J. Threat. Taxa. 2015; 7: 7309–7408.

163. Vanderplank SE. The Vascular Flora of Greater San Quintín, Baja California, Mexico. CGU Theses & Dissertations; 2010.

164. Pandža M. Flora parka prirode Papuk (Slavonija, Hrvatska). Šumarski list. 2010; 134: 25–43.

165. Tropicos. Paraguay Checklist. Tropicos. St. Louis: Missouri Botanical Garden; 2015.

166. Kattge J, Diaz S, Lavorel S, Prentice IC, Leadley P, Bönisch G, et al. TRY – a global database of plant traits. Global Change Biology. 2011; 17: 2905–2935.

167. Gamit SB, Maurya RR, Qureshimatva UM, Solanki HA. Check list of flowering plants in Tapi District, Gujarat, India. International Journal of advanced Research. 2015; 3: 1104–1123.

168. University of Greifswald. FloraGREIF - Virtual Flora of Mongolia; 2010. Available: http://greif.uni-greifswald.de/floragreif/. Accessed 2 February 2016.

169. Letcher SG, Lasky JR, Chazdon RL, Norden N, Wright SJ, Meave JA, et al. Environmental gradients and the evolution of successional habitat specialization. A test case with 14 Neotropical forest sites. J Ecol. 2015; 103: 1276–1290. doi: 10.1111/1365-2745.12435.

170. Oliveira-Filho AT. NeoTropTree. Flora arbórea da Região Neotropical: Um banco de dados envolvendo biogeografia, diversidade e conservação; 2014. Available: http://www.icb.ufmg.br/treeatlan/. Accessed 12 February 2016.

171. Schaefer H, Hardy OJ, Silva L, Barraclough TG, Savolainen V. Testing Darwin's naturalization hypothesis in the Azores. Ecol Lett. 2011; 14: 389–396. doi: 10.1111/j.1461-0248.2011.01600.x.

172. Royal Botanic Gardens Kew. Seed information database (SID). v7.1; 2016. Available: http://data.kew.org/sid/. Accessed 22 March 2013.

173. Benito BM, Lorite J, Pérez-Pérez R, Gómez-Aparicio L, Peñas J, Robertson M. Forecasting plant range collapse in a mediterranean hotspot. When dispersal uncertainties matter. Diversity Distrib. 2014; 20: 72–83. doi: 10.1111/ddi.12148.

174. Kraft TS, Wright SJ, Turner I, Lucas PW, Oufiero CE, Supardi Noor MN, et al. Seed size and the evolution of leaf defences. J Ecol. 2015; 103: 1057–1068. doi: 10.1111/1365-2745.12407.

175. Lopez-Martinez JO, Sanaphre-Villanueva L, Dupuy JM, Hernandez-Stefanoni JL, Meave JA, Gallardo-Cruz JA. Beta-Diversity of functional groups of woody plants in a tropical dry forest in Yucatan. PLoS One. 2013; 8: e73660. doi: 10.1371/journal.pone.0073660.

176. Zhang ST, Zhen Du G, Chen JK. Seed size in relation to phylogeny, growth form and longevity in a subalpine meadow on the east of the Tibetan plateau. Folia Geobot. 2004; 39: 129–142. doi: 10.1007/BF02805242.

177. Engemann K, Sandel B, Boyle BL, Enquist BJ, Jørgensen PM, Kattge J, et al. A plant growth form dataset for the New World. Ecology. 2016; 97: 3243. doi: 10.1002/ecy.1569.

178. Jiménez JE, Juárez P, Díaz A. Checklist of the vascular flora of Reserva Biológica San Luis, Costa Rica. Check list. 2016; 12: 1859. doi: 10.15560/12.2.1859.

179. Da Vela M, Frignani F, Bonari G, Angiolini C. La flora vascolare della diserva naturale "La Pietra" (Toscana meridionale). Micol. Veget. Medit. 2013; 28: 135–160.

180. Paula S, Arianoutsou M, Kazanis D, Tavsanoglu Ç, Lloret F, Buhk C, et al. Fire‐related traits for plant species of the Mediterranean Basin. Ecology. 2009; 90: 1420.

181. Wieringa JJ. Flora of Gabon. unpublished; 23.04.2016.

182. Ter Steege H, Vaessen RW, Cardenas-Lopez D, Sabatier D, Antonelli A, Oliveira SM de, et al. The discovery of the Amazonian tree flora with an updated checklist of all known tree taxa. Sci Rep. 2016; 6: 29549. doi: 10.1038/srep29549.

183. Heberling JM, Jo I, Kozhevnikov A, Lee H, Fridley JD. Biotic interchange in the Anthropocene. Strong asymmetry in East Asian and eastern North American plant invasions. Global Ecol. Biogeogr. 2017; 26: 447–458. doi: 10.1111/geb.12551.

184. Turner IM. A catalogue of the vascular plants of Malaya. Singapore: Gardens' Bulletin; 1995.

185. BGCI. GlobalTreeSearch online database; 2017. Available: www.bgci.org/globaltree_search.php. Accessed 14 August 2017.
